# Mitochondrial Targeting and Imaging with Small Organic Conjugated Fluorophores: A Review

**DOI:** 10.1002/chem.202202366

**Published:** 2022-10-26

**Authors:** Hannah Crawford, Maria Dimitriadi, Jatinder Bassin, Michael T. Cook, Thais Fedatto Abelha, Jesus Calvo‐Castro

**Affiliations:** ^1^ School of Life and Medical Sciences University of Hertfordshire AL109AB Hatfield UK; ^2^ Department of Pharmacology, Toxicology and Therapeutic Chemistry Faculty of Pharmacy and Food Science University of Barcelona 08028 Barcelona Spain; ^3^ Institute of Nanoscience and Nanotechnology University of Barcelona (IN2UB) 08028 Barcelona Spain

**Keywords:** bioimaging, fluorescence, mitochondria, optoelectronics, organic materials

## Abstract

The last decade has seen an increasingly large number of studies reporting on the development of novel small organic conjugated systems for mitochondrial imaging exploiting optical signal transduction pathways. Mitochondria are known to play a critical role in a number of key biological processes, including cellular metabolism. Importantly, irregularities on their working function are nowadays understood to be intimately linked to a range of clinical conditions, highlighting the importance of targeting mitochondria for therapeutic benefits. In this work we carry out an in‐depth evaluation on the progress to date in the field to pave the way for the realization of superior alternatives to those currently existing. The manuscript is structured by commonly used chemical scaffolds and comprehensively covers key aspects factored in design strategies such as synthetic approaches as well as photophysical and biological characterization, to foster collaborative work among organic and physical chemists as well as cell biologists.

## Graphical abstract

**A comprehensive overview** on reported small molecule fluorophores exploited as mitochondrial imaging materials is carried out, with particular focus on the synthetic approaches used for the different chemistries, the photophysical characteristics of the probes and their cell biology evaluation.

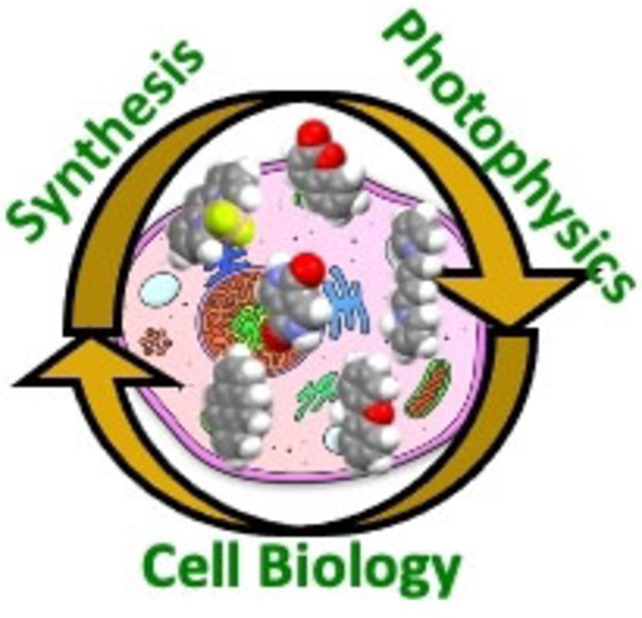

## Introduction

**Figure 1:**
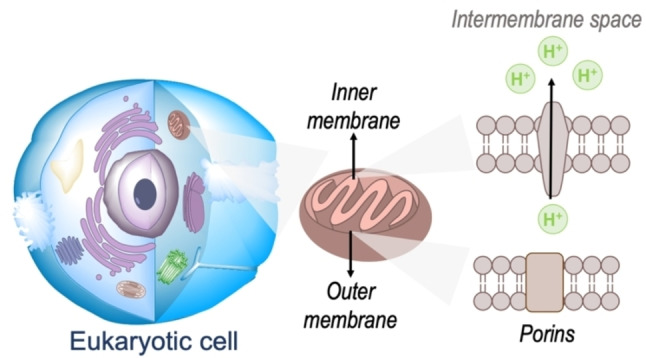
Representation of a eukaryotic cell highlighting the mitochondria in the cytoplasm as well as the structure of the inner and outer membranes.

The realization of novel biomaterials with specific subcellular localization is at the forefront of current research interests and efforts.[^1^, ^2^, ^3^, ^4^, ^5^] Among those different organelles, mitochondria have attracted an increasingly large surge of interest in recent years with more than 70 % of all novel molecules reviewed in this work published in the last five years. Mitochondria denote a cytoplasmic organelle that has likely evolved from the incorporation of bacteria within eukaryotic cells through endosymbiosis and, importantly, have their own circular DNA.[^6^, ^7^] Structurally, they present variable shapes (i. e., spherical to oval), numbers and sizes (e. g., 0.5–3 μm) in different cells and are constituted by a double membrane of different permeability.[^8^, ^9^, ^10^] The smooth outer membrane regulates the entrance of small molecules (up 5000 Da) through channels of porin proteins (Figure 1), also referred to as the voltage dependent anion channels (VDAC).^[11]^ In turn, the inner membrane, which is thinner (thickness=ca 5 and 7 nm for the inner and outer mitochondrial membranes, respectively) presents parallel infoldings of cristae attached to it.^[12]^ The abovementioned increasing interests in the development of biomaterials with specific mitochondrial localization can be readily associated to their roles in energy generation, calcium storage as well as regulation of cellular metabolism and ATP production. Abnormalities in the generation of reactive oxygen species during the latter are often associated to oxidative stress and apoptosis.[^13^, ^14^, ^15^] Importantly, it is nowadays widely accepted that a number of pathological conditions, including cancer, autoimmune disorders and neurodegenerative disorders such as Alzheimer's and Parkinson's diseases, are linked to oxidative damage and mitochondrial malfunction.[^16^, ^17^, ^18^, ^19^] As a result of this, the development of novel imaging strategies for this subcellular organelle is highly desired. Whilst magnetic resonance imaging, radioisotope labelling and positron emission tomography have all been previous exploited as bioimaging techniques for monitoring subcellular organelles, these suffer from drawbacks such as high operational costs, organelle degradation and isotope effects which highly warrant the development of alternative methodologies.[^1^, ^20^, ^21^, ^22^, ^23^] More recently, approaches exploiting fluorescence emission of small organic conjugated molecules have emerged as a promising alternative class of imaging strategies.[^1^, ^24^, ^25^, ^26^] This is largely based on their ability to translate molecular recognition into highly discriminative and easily detected optical signals.

In engineering novel small organic π‐conjugated bio‐optoelectronic materials, one can consider two main approaches: (i) the widely‐exploited method of performing (often) judicious peripheral substitutions on known core motifs for which successful performance has been previously observed and (ii) the realization of novel core platforms with views to outperform currently existing chemistries that can increase the toolbox of available materials. In specifically targeting the mitochondria, the most widely‐used approach is the exploitation of mitochondria's highly negative transmembrane potential; this is used during respiration processes whereby proton pumps located in the inner mitochondrial membrane transport protons onto the intermembrane space (Figure 1).[^27^, ^28^, ^29^, ^30^] Thus, design strategies are largely based on employing delocalized lipophilic cations (DLCs) such as triphenylphosphonium and pyridinium cation as peripheral substituents to otherwise neutral core motifs.[^1^, ^2^, ^3^, ^31^, ^32^, ^33^] However, the use of positively charged moieties carry drawbacks which are often overlooked. In short, these cationic species can reduce the membrane potential and negatively impact on the critical working function of the organelle. Importantly, probe leakage through the membrane due to a decrease in the potential can lead to a decrease in the efficiency of the staining agents.[^2^, ^31^] In response to the these, the development of molecules that retain preferential mitochondrial accumulation whilst being neutral represent a major innovation in the field and are highly warranted.[^34^, ^35^]

**Figure 2:**
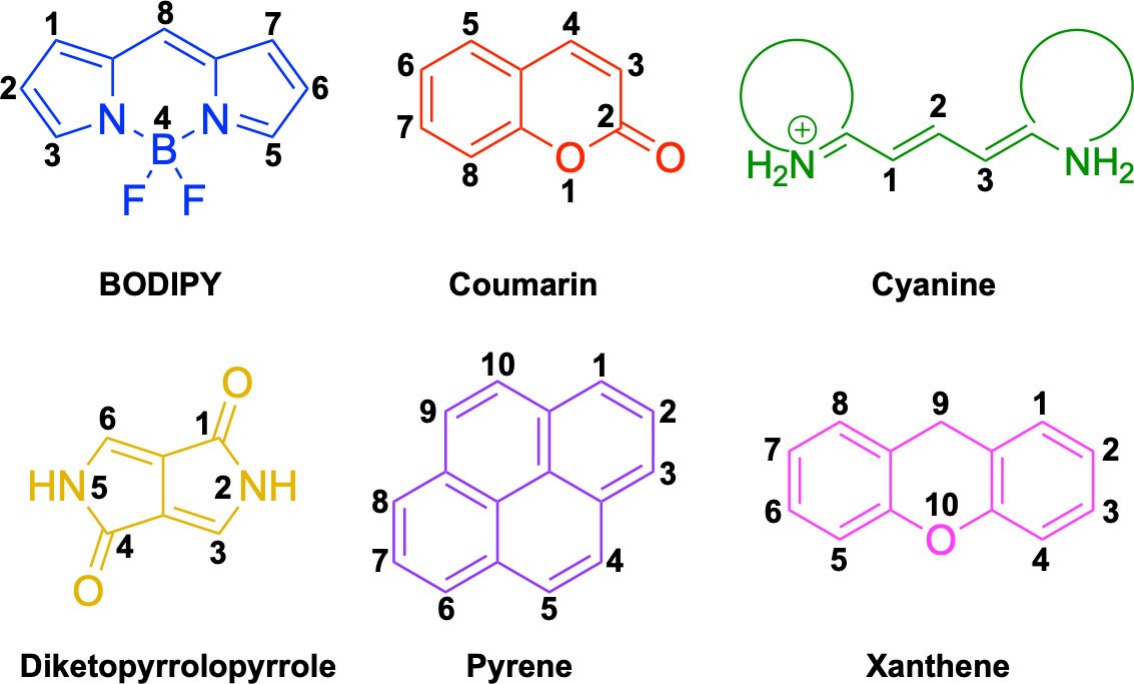
Chemical structures and IUPAC numbering for commonly used fluorescent core motifs for mitochondrial imaging.

In this work, we carry out a comprehensive overview of the different chemistries that have been exploited to date for the realization of small organic fluorophores exhibiting preferential mitochondrial accumulation. It should be noted that reports where such core motifs are utilized as building blocks in polymeric materials or ligands in metal complexes or bear significant structural alterations to an extent that the properties of the molecules do not resemble those of the key core chemistry are not within the remit of this review. Similarly, so‐called hybrid materials encompassing the photophysical properties of more than one core motif were not reviewed in this work.[^36^, ^37^, ^38^, ^39^, ^40^, ^41^, ^42^, ^43^] In the case of small molecules whose intended purpose or application was different (e. g., photodynamic therapy) whilst exhibiting mitochondrial accumulation, these were included for completeness and to inform future design strategies. First, we make use of the well‐known Jablonski diagram to provide a comprehensive overview of the different photophysical processes upon light absorption and identify sought‐after characteristics that can serve to design judicious approaches. In this regard, a factor often disregarded in design approaches is the available instrumentation in laboratory settings (e. g., fluorescence microscope channels), which can sometimes represent a limitation in the evaluation of novel materials. Subsequently, main chemistries utilized for the development of small organic fluorophores for this subcellular organelle (Figure 2), such as BODIPYs, coumarins, cyanines, diketopyrrolopyrroles, pyrenes and xanthenes are each revised in detail, including, to the best of our knowledge, all reported molecules to date bearing these chemical scaffolds. As a result, we anticipate this review paper to enhance multidisciplinary awareness among organic and physical chemists as well as cell biologists and to further foster collaborative work to pave the way for the discovery of superior mitochondrial fluorescent imaging platforms.

## Photophysical processes in organic molecules

**Figure 3:**
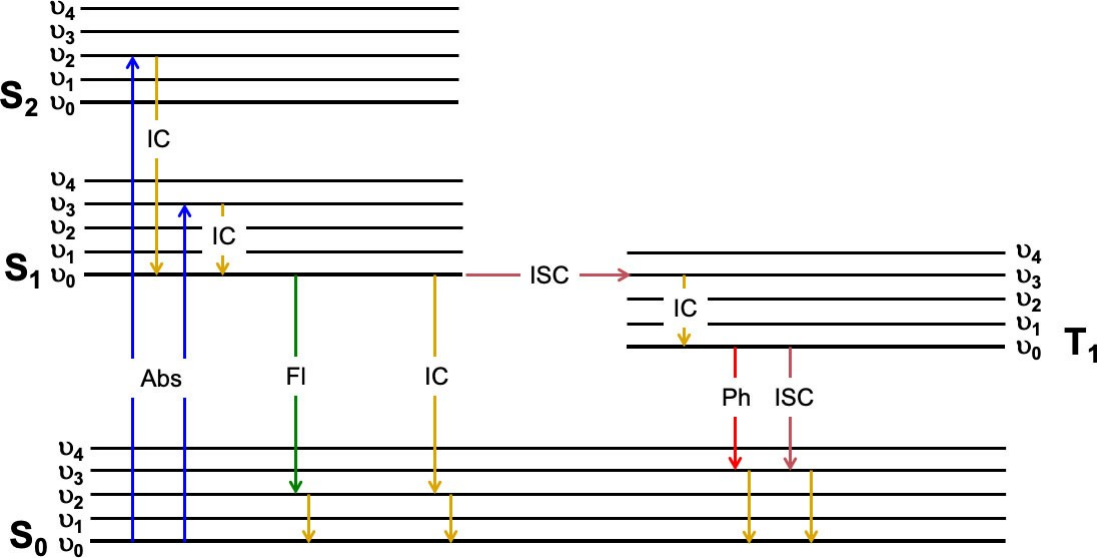
Simplified Jablonski diagram illustrating most common photophysical processes in organic molecules.

The simplified Jablonksi diagram in Figure 3 illustrates the most common electronic transitions that occur in organic molecules, where singlet and triplet states are conventionally aligned to the left and right, respectively, and main energy levels are subdivided into vibrational energy levels (note that rotational energy levels are not illustrated for simplicity). Upon light absorption, chromophores (i. e., molecules that absorb light) are promoted to a vibrational energy level of a higher energy singlet excited state (S_n>0_). As illustrated in Figure 3, this does not necessarily mean the first singlet excited state (S_1_) if sufficient energy is provided. Two parameters are key in characterizing a chromophore: (i) the absorption maximum, λ_abs_^max^ (i. e., the wavelength at which the molecule absorbs the most light) and (ii) the absorption factor or coefficient, ϵ which determines the ability of the molecule to absorb light of a particular wavelength. Note that the latter is generally referred to molar absorption factor or coefficient since the concentrations of working solutions are often given in units of molarity. It is noteworthy that these properties are solvent dependent and care must be taken when assuming negligible changes in cell media when compared to commonly used organic solvents. In addition, lower solubility in aqueous media can lead to fluorescence quenching due to newly accessible deactivation pathways available through aggregation.

In most cases, the aim is to structurally modify materials so these chemical alterations result in red or bathochromic (i. e., longer wavelength) shifts. This is associated to longer/lower energy excitation wavelengths being consistent with greater tissue penetration, reduced background emission as well as lower probability of induced side reactions as a result of the higher energy such as photobleaching.^[25]^ Structurally, bathochromic shifts in the absorption spectrum can be achieved through enhancing conjugation (i. e., electron delocalization) as well as inducing greater rigidity through conjugated backbones by, for example, limiting free rotation of peripheral substituents. However, such strategies also bear associated drawbacks in the performance of these materials (see below).

Irrespective of the energy and vibrational energy level to which molecules are promoted upon excitation, next, molecules relax to the lowest vibrational energy level of the first singlet excited state (S_1_) through a process known as internal conversion (IC) since there is not a spin change as a result of this transition. This is known as Kasha's Rule and as a consequence, fluorescence emission spectral profiles are independent of the excitation wavelength.^[44]^ In all cases, excited states are unstable, meaning that the molecule will return back to the ground state. However, from the lowest vibrational energy level of S_1_ this can take place through different photophysical mechanisms as illustrated in Figure 3; radiatively in a process known as fluorescence emission or radiationless by internal conversion (no change in spin configuration under the assumption that the most common case for organic conjugated materials is that of a closed‐shell, singlet electronic ground state, S_0_). In this regard, three parameters are critical in characterizing a fluorophore (i. e., a molecule that emits light as fluorescence), namely fluorescence quantum yield, φ_f_, fluorescence emission maximum, λ_em_^max^, and fluorescence lifetime, τ_f_. The latter denotes the time that a fluorophore remains in the excited state and can be expressed as the inverse of the sum of radiative and radiationless (or non‐radiative) decay rate constants, k_r_ and k_nr_ respectively. Whilst it is not often considered as an important parameter in the bioimaging field, it should be noted that small lifetimes result in lower probability of the excited states, which are intrinsically more reactive that ground states, undergoing other secondary processes.

The fluorescence quantum yield denotes the number of photons emitted as fluorescence with respect to the total number of photons absorbed. It can also be expressed as the ratio of k_r_ with respect to the sum of k_r_ and k_nr_. Although large fluorescence quantum yields are highly desired in fluorescence bioimaging, so‐called turn‐on bifunctional probes, which are designed for in situ ratiometrically reporting of target analytes, often exploit the concept of photoinduced electron transfer.[^25^, ^45^] In such systems, fluorescence is intrinsically quenched via this mechanism resulting in low φ_f_. Interaction of the fluorophore with the target analyte disrupts the quenching event and increases fluorescence emission allowing for the transduction of the detection. This aside, novel materials not intended as bifunctional probes should not be solely discarded on the basis of preliminary and arguably low quantum yields. Instead, the brightness of the material, which can be denoted as the product of the absorption factor (see above) and the fluorescence quantum yield should be evaluated. In the bioimaging field, materials exhibiting large brightness are highly sought due to the lower experimental concentrations required, and hence alleviating potential cell viability concerns. In addition, high brightness also aids in cases where developed materials exhibit poor water solubility and a widely‐used design strategy is to develop materials highly soluble in dimethyl sulfoxide (DMSO). This can be used as a vehicle solvent since concentrations of this solvent lower than 1 % v/v are known to induce minimal cell viability issues.[^46^, ^47^, ^48^] Although care must be taken into the design of novel molecules to influence the probability of radiative vs. radiationless decay transitions, application of the Fermi Golden rule of perturbation theory and Equations derived from the Energy Gap Law for radiationless transitions result in a decrease of k_nr_ as the optical gap^[49]^ increases.[^50^, ^51^] In short, the larger the energy required to be dissipated on going from excited to ground state, the less likely this will take place via radiationless processes. Importantly, this represents a challenge in the design of materials for fluorescence bioimaging as well as other applications benefiting from longer excitation wavelengths. If longer wavelengths are achieved as a consequence of rigid core motifs and/or conjugated backbones, these will carry the associated drawback for bioimaging of small Stokes shifts (i. e., difference between absorption and fluorescence emission maxima), since the restricted flexibility of the molecule results in very small possible structural re‐arrangements upon photon absorption. Small Stokes shifts are associated with self‐absorption which will ultimately negatively impact on the performance of the material. For example, in diketopyrrolopyrroles the choice of core rings can significantly impact these photophysical aspects.^[52]^

Alternatively, a molecule in the lowest vibrational energy level of S_1_ can undergo a classically, within the zero or pure Born‐Oppenheimer approximation, spin forbidden transition to an upper vibrational energy level of the first triplet excited state (T_1_). This transition, which is often forgotten to be isoenergetic in simplified Jablonski diagrams (i. e., the interaction of vibrational energy levels of different multiplicity requires them to be degenerated), is known as intersystem crossing and requires a change in spin configuration unlike internal conversion. It is noteworthy that non‐radiative decay rate constants, used in the determination of fluorescence lifetimes and quantum yields, do consider both internal conversion and intersystem crossing processes. To a first approximation, these transitions become allowed through a spin mixing of states involved, which in turn arises from magnetic interaction, so‐called spin‐orbit coupling.[^25^, ^53^] Assuming a purely atomic interaction, the strength of it increases with the fourth power of the atomic number. Thus, a ubiquitous approach is to promote this effect by halogen substitutions which can enhance the torque of the electrons. For the purpose of this work, it should be noted, however, that not all halogen‐bearing scaffolds will exhibit lower fluorescence quantum yields as a result of increased triplet excited state population through intersystem crossing. In fact, in order to induce this, the peripheral heavy atom substitutions must be aligned with the dipole moment for the transition. Whilst this alternative deactivation process is detrimental for maximizing the fluorescence quantum yield, it does have interesting technologically relevant applications such as in photodynamic therapy. Once at the lowest vibrational energy level of T_1_, the molecule can return to the ground state via radiative (phosphorescence) or radiationless processes.

## BODIPYs

Boron dipyrromethene difluoride (BODIPY, Figure 2) derivatives have been widely utilized in optoelectronic applications on account of their desirable properties, such as high brightness and photostability as well as the versatility of the core motif.^[54]^ Despite their intrinsic hydrophobicity, which could make them to be discarded for bioapplications associated to polar organelles, there is a significant number of studies exploiting BODIPY chemistries that exhibit preferential mitochondrial accumulation.

Synthetically, the way this class of materials is afforded is dictated by the overall symmetry of the molecule. Symmetric systems[^34^, ^55^, ^56^, ^57^, ^58^, ^59^, ^60^, ^61^, ^62^, ^63^, ^64^, ^65^, ^66^, ^67^] can be accessed by reaction of the desired substituted pyrrole with an aldehyde or an acid chloride/anhydride.[^68^, ^69^, ^70^, ^71^, ^72^] In both cases, the first synthetic step is the generation of the relevant dipyrromethene intermediate.^[69]^ In cases where the approach of choice is the use of aldehydes, the reaction proceeds via an acid‐catalyzed condensation to form the intermediate which then requires direct oxidation due to its instability.^[69]^ The final symmetrical BODIPY is accessed by reacting the oxidized intermediate with a source of BF_2_ under basic conditions.^[73]^ If the synthetic route involves the use of acid chloride/anhydride, the dipyrromethene intermediate is directly afforded and then reacted with a source of BF_2_ to yield the final BODIPY of choice.[^68^, ^70^, ^71^] Alternatively, the first part of the synthetic protocol can be carried out by employing phosphorous oxytrichloride in the self‐condensation reaction of formylated pyrrole derivatives.^[74]^ Along these lines, asymmetric architectures[^34^, ^58^, ^59^, ^60^, ^61^, ^63^, ^64^, ^65^, ^75^, ^76^, ^77^, ^78^, ^79^, ^80^, ^81^] can be accessed following the latter with the addition of a second pyrrole with the desired substitution.[^54^, ^82^] It is noteworthy that a synthetic strategy to affording NIR materials bearing the BODIPY scaffold is through fused aromatic substitutions that result in a newly expanded core structure.[^83^, ^84^] However, we consider these not to be within the scope of this review article.

Peripheral substitutions on positions 3 and 5 of the core motif are ubiquitous, as are those on position 8, the so‐called meso position. Substitutions on the former are often associated with inducing bathochromic spectral shifts with respect to those observed for the unsubstituted parent motif. However, there are reports (**1**–**3**, Table 1) on tuning the fluorescence emission efficiency by substitutions on these positions, with observations made consistent with larger quantum yields for mono substitutions (φ_f_=0.42 and 0.21 for **2** and **3** in acetonitrile, respectively).^[34]^ In turn, substitutions on the meso position are known to have a greater effect on the photophysical and electrochemical properties of BODIPY systems.^[67]^ This can be readily associated to the distinctly different nodal progression of the frontier molecular orbitals in BODIPY‐based architectures. In short, whilst the meso carbon atom is characterized by a node in the HOMO surface, a large density is observed in the surface of the frontier molecular orbital counterpart.[^66^, ^79^, ^85^] Nonetheless, to exert a significant effect on the photophysical properties of the system, such peripheral alterations must be conjugated to the core motif. Importantly, substitutions on this position have been reported to often lead to alternative radiationless deactivation pathways with the associated detrimental effect of diminishing the fluorescence quantum efficiency (e. g., φ_f_=0.63, 0.33 and 0.04 for **4**, **5** and **6** in phosphate buffered saline (PBS), respectively).[^65^, ^67^, ^75^] This can be alleviated via ortho‐substitution, resulting in out of plane re‐arrangement of the meso substituents with respect to the planar BODIPY core motif (φ_f_=0.88 for **7** in acetonitrile).^[78]^

**Table 1:** Chemical structure, absorption and fluorescent emission maxima (λ_abs/em_^max^/nm), Stokes shifts (Δλ/cm^−1^), fluorescence quantum yield (φ_f_) and solvent use for the characterization of reported BODIPY‐based architectures.

Molecule	Chemical structure	λ_abs/em_^max^/nm	Δλ/cm^−1^	φ_f_	Solvent
**1**	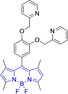	498/510	472	0.51	Acetonitrile
**2**	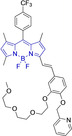	570/589	565	0.42	Acetonitrile
**3**	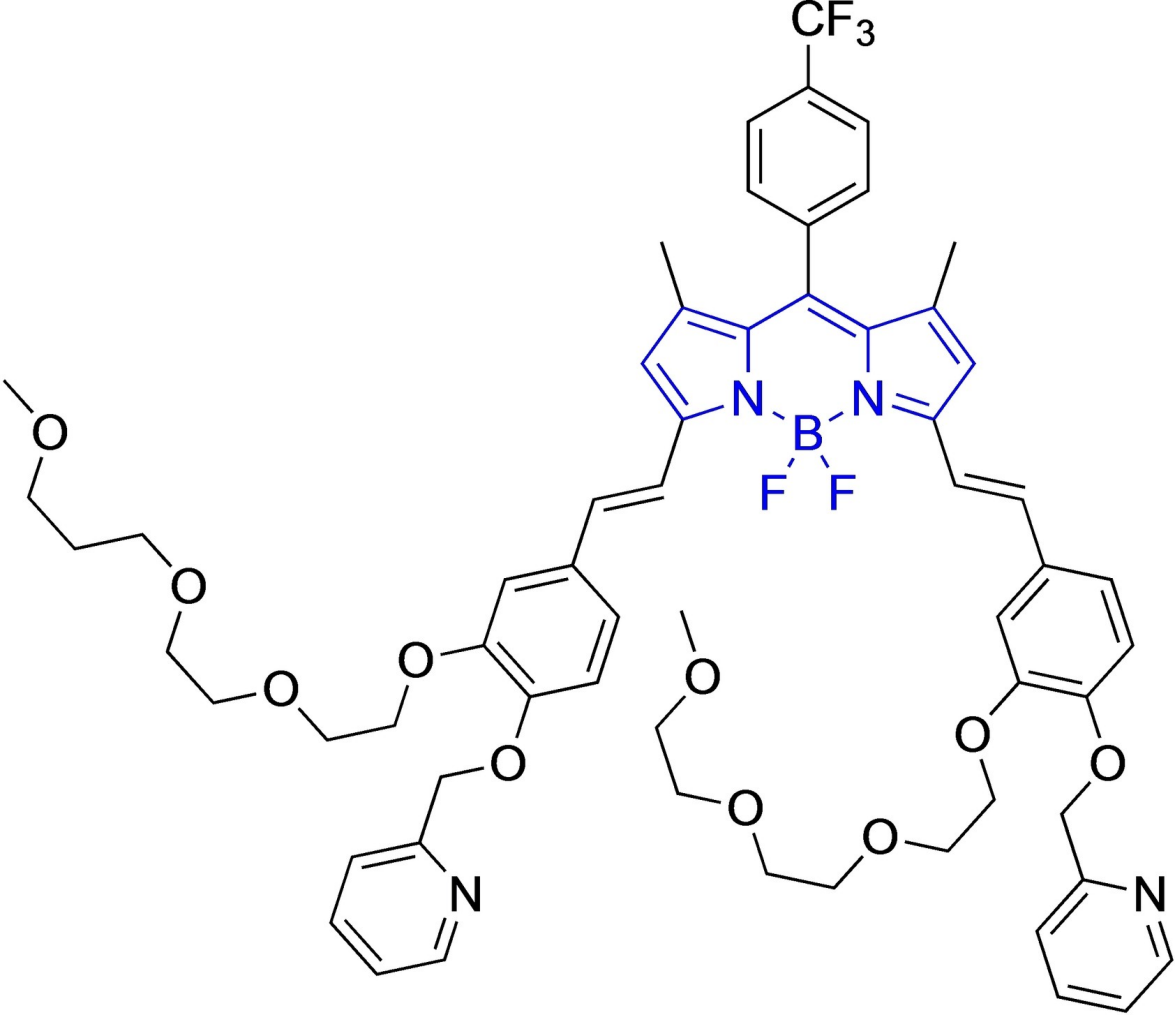	645/666	488	0.21	Acetonitrile
**4**	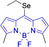	532/548	548	0.63	PBS
**5**	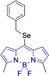	530/545	519	0.33	PBS
**6**	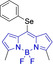	532/550	615	0.04	PBS
**7**	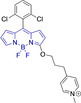	501/526	948	0.88	Acetonitrile
**8**	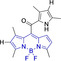	513/548	1245	0.01	DCM
**9**	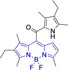	524/573	1631	0.01	DCM
**10**	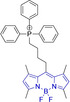	499/515	622	1.00	Ethanol
**11**	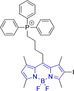	510/528	668	0.17	Ethanol
**12**	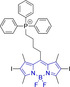	528/544	557	0.08	Ethanol
**13^[a]^**	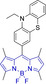	500/515	582	–	PBS (50 % ethanol)
**14**	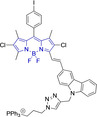	612/650	955	0.40	Ethanol
**15**	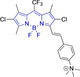	618/625	181	0.03	Water
**16**	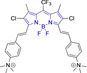	679/703	502	0.01	Water
**17**	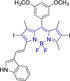	636/676	930	0.17	DMSO
**18**	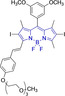	601/624	613	0.52	DMSO
**19**	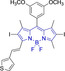	604/621	453	0.15	DMSO
**20**	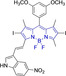	622/651	716	0.19	DMSO
**21**	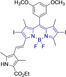	612/639	690	0.23	DMSO
**22**	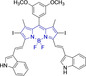	727/771	784	0.10	DMSO
**23**	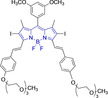	666/695	626	0.17	DMSO
**24**	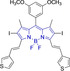	675/692	363	0.01	DMSO
**25**	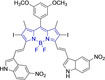	712/748	675	0.02	DMSO
**26**	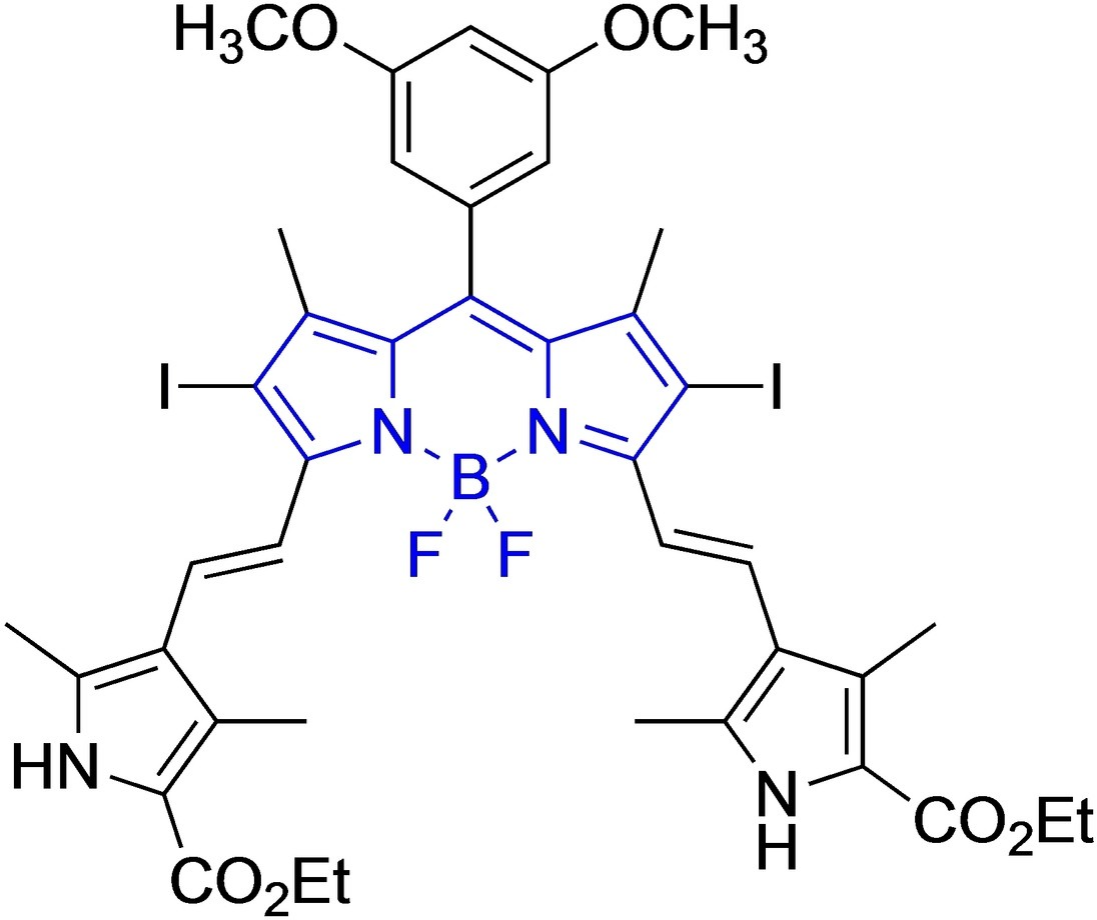	693/731	750	0.07	DMSO
**27**	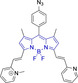	639/674	812	‐	DMSO
**28**	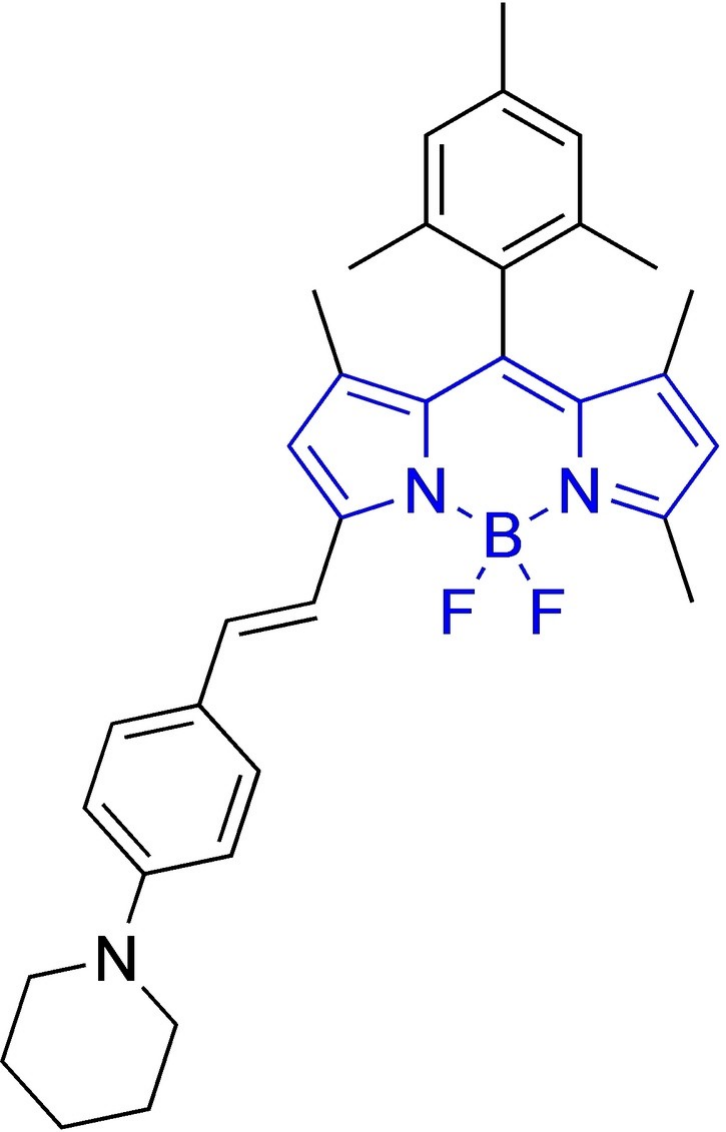	588/707	2862	0.06	Acetonitrile
**29**	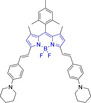	681/751	1368	0.04	Acetonitrile
**30**	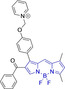	504/594	3006	–	DMSO
**31**	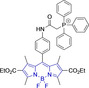	505/510	194	0.30	PBS (1 % DMSO)
**32**	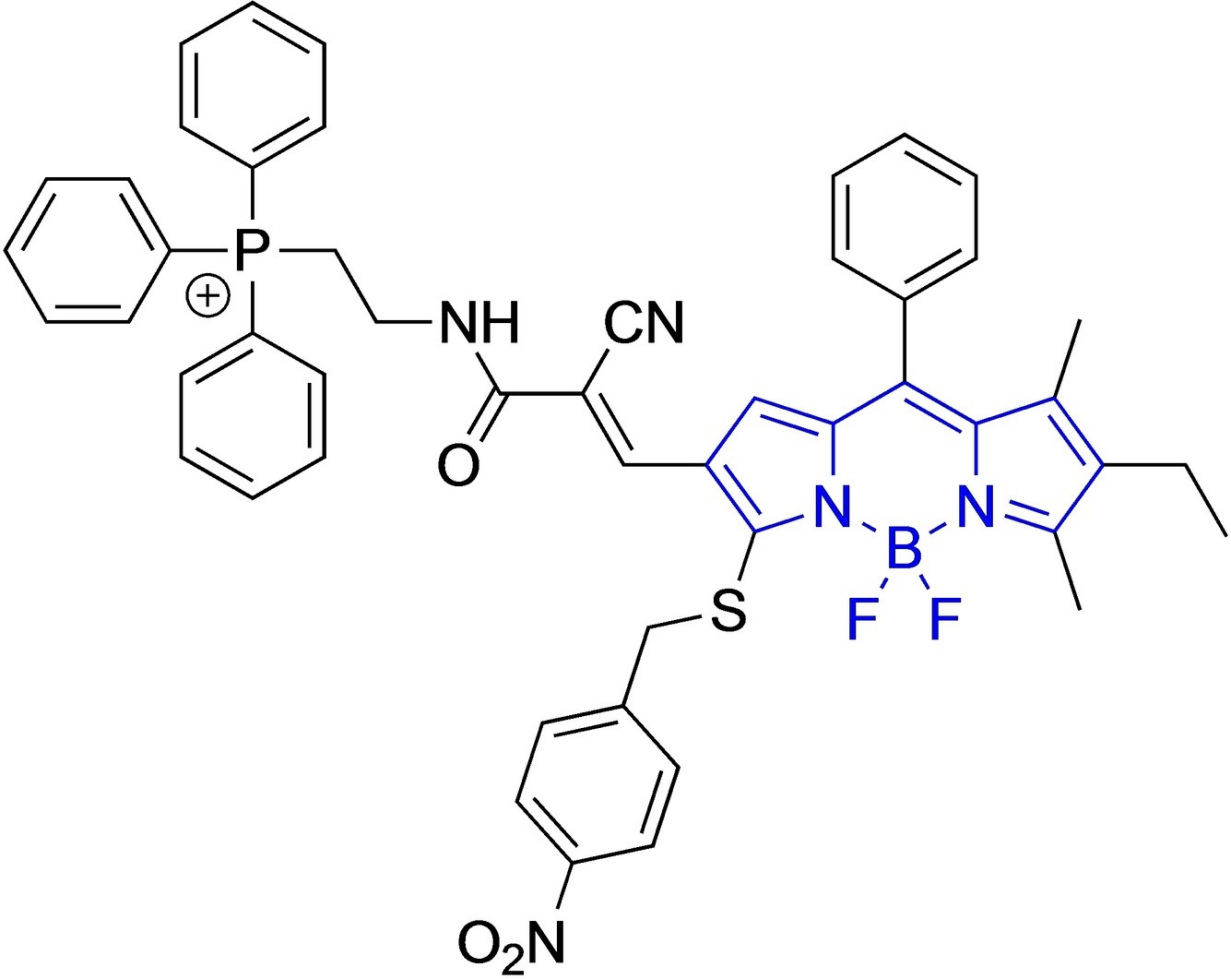	495/560	2344	0.18	DMSO/Tris‐HCL 1 : 3 v/v
**33**	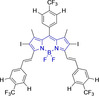	638/662	568	0.17	DMF
**34**	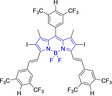	640/661	496	0.16	DMF
**35^[a]^**	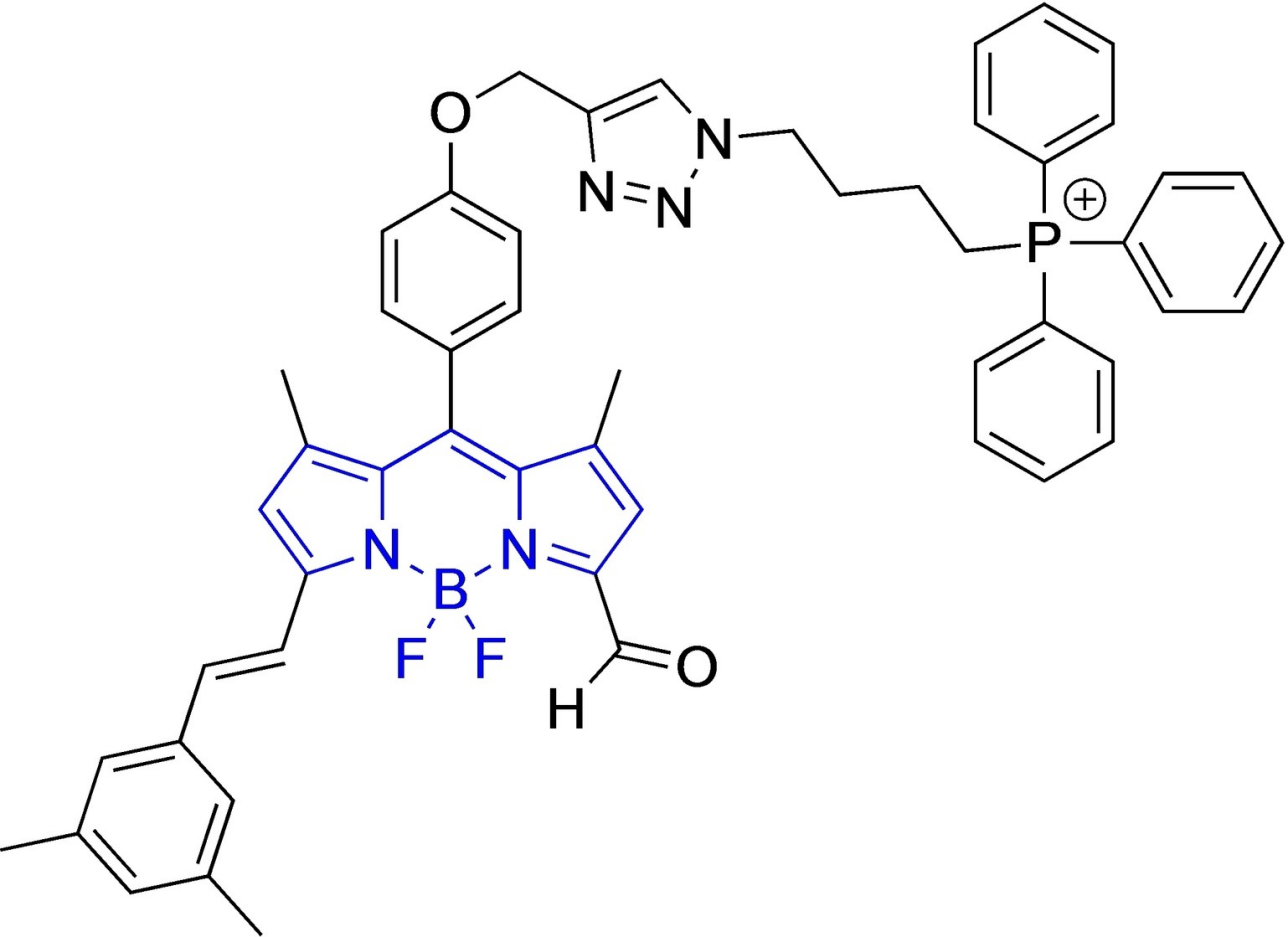	–/590	–	–	
**36**	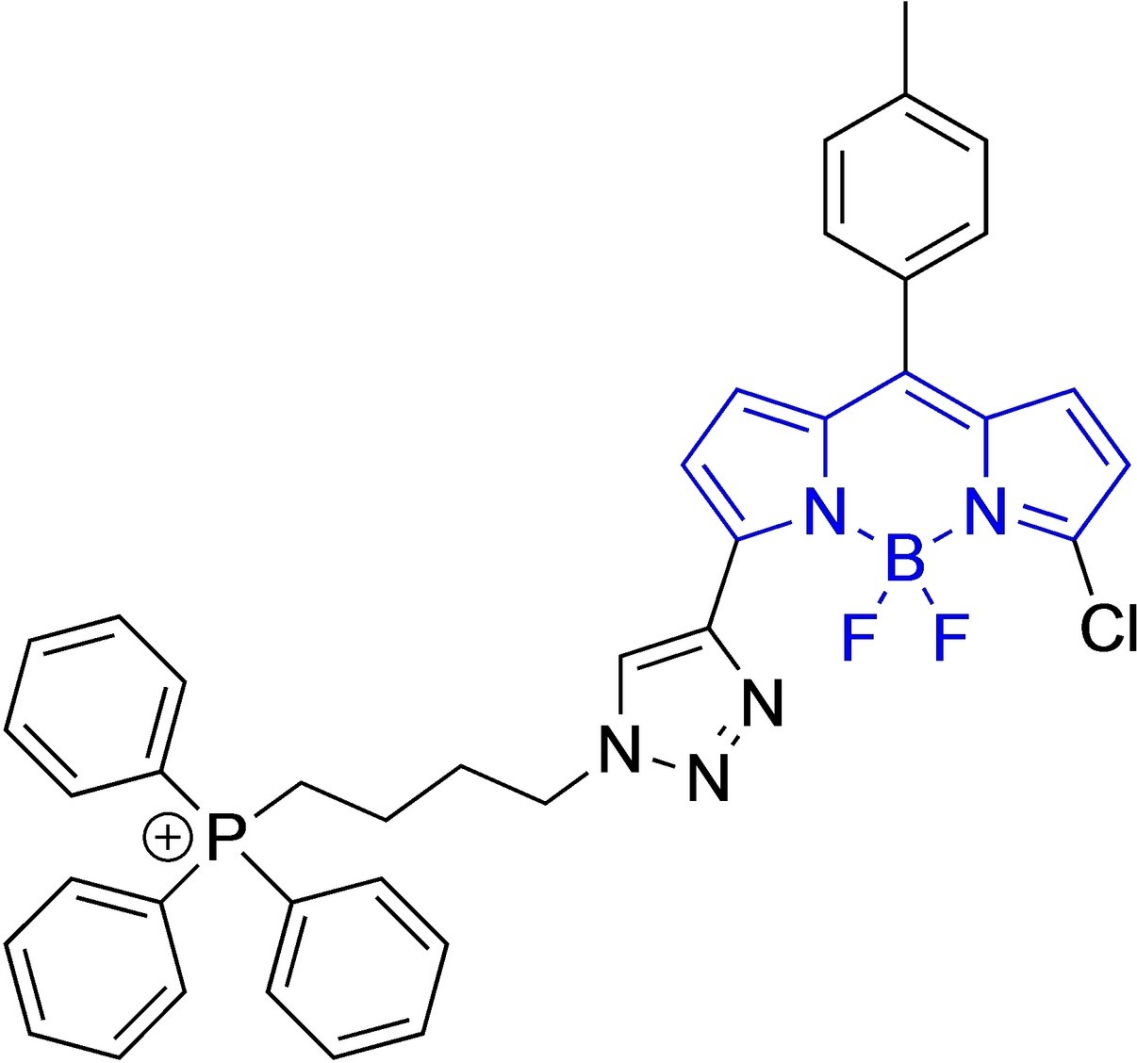	540/557	565	–	Acetonitrile/HEPES buffer (5 : 95)
**37**	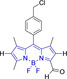	491/516	986	0.02	DMSO
**38**	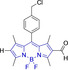	498/511	510	0.40	DMSO
**39**	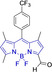	491/509	720	0.17	DMSO
**40**	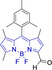	505/511	232	0.19	DMSO
**41**	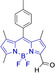	493/507	560	0.24	DMSO
**42**	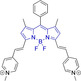	647/679	728	0.1	PBS (1 % DMSO)
**43**	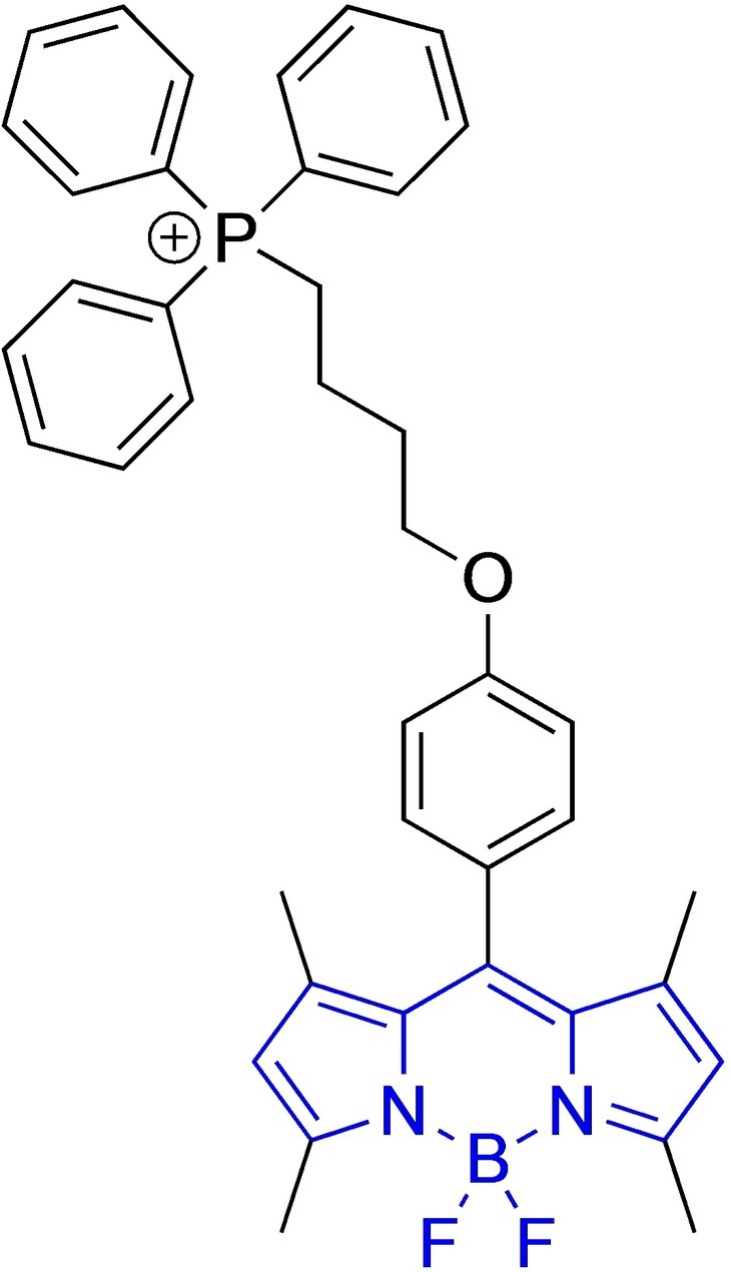	496/509	514	0.49	Water
**44**	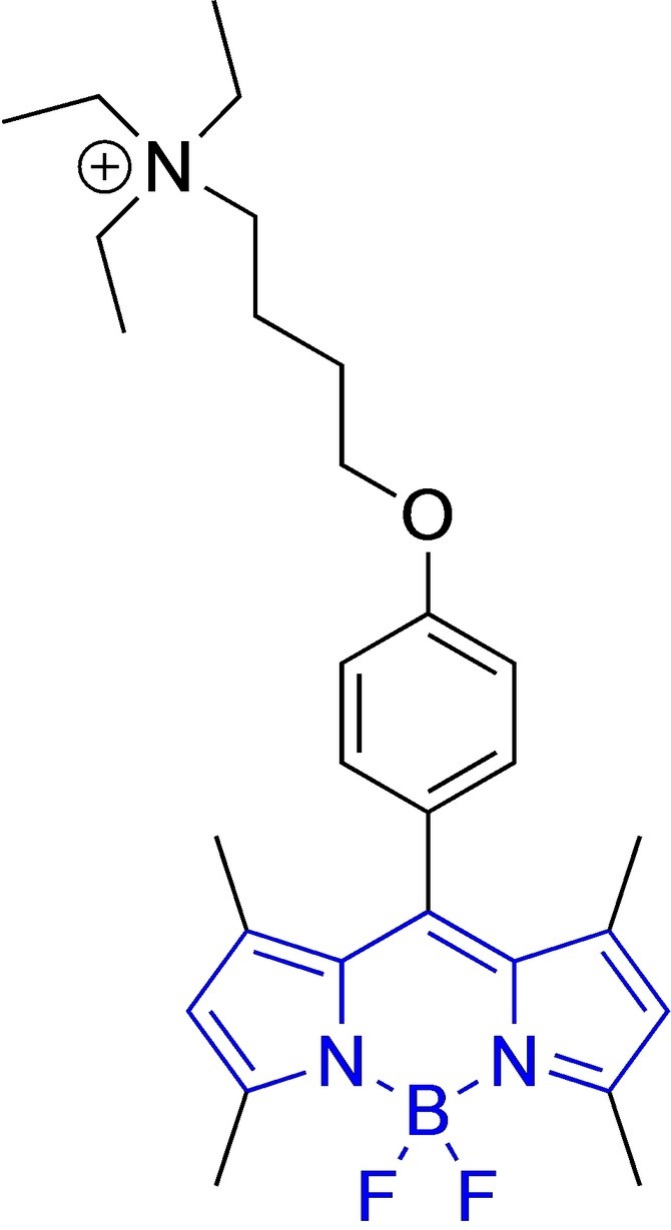	496/508	476	0.69	Water
**45**	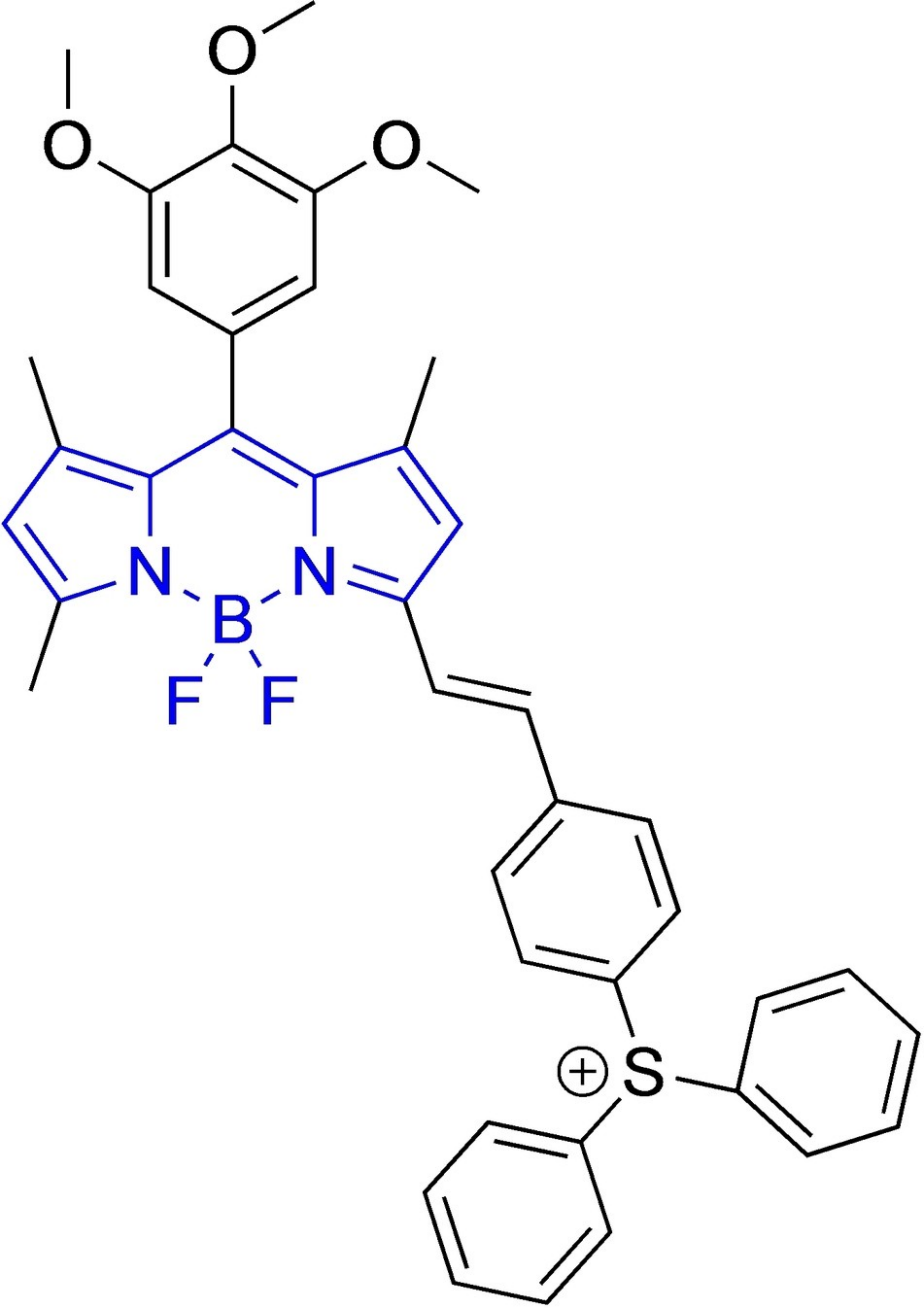	565/580	457	–	–
**46**	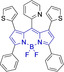	635/661	619	0.52	DMSO
**47**	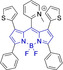	665/706	873	0.26	DMSO

^[a]^ Absorption and fluorescence emission maxima approximated from spectra.

Whilst such approaches would limit the bathochromic effect normally associated to meso substitutions, impaired rotation ensures large quantum efficiencies. Subtle peripheral alterations, such as simultaneous H for Me on the ketopyrrolyl meso and position 2 in **8** and **9**, respectively, can lead to bathochromic changes (λ_em_^max^=548 and 573 nm for **8** and **9** in dichloromethane (DCM), respectively) whilst maintaining intentionally sought negligible quantum yields.^[75]^ In some cases, such low quantum efficiencies are judiciously designed to access probes able to ratiometrically report on the concentration of a particular mitochondrial analyte by changes in their fluorescence intensity.^[55]^ Although this can be achieved through heavy atom substitutions on positions 2 and/or 6 (e. g., φ_f_=1.00, 0.17, 0.08 for **10**, **11** and **12** in ethanol, respectively),[^59^, ^60^, ^65^] it is primarily accessed through intramolecular photoinduced electron transfer processes such as in a neutral phenothiazine‐containing BODIPY (**13**). Lower fluorescence quantum yields have also been reported for carbazole‐bearing BODIPYs with p‐iodine substitution on the meso position (φ_f_=0.40 for **14** in ethanol).^[79]^ Judicious meso‐substitutions have been explored in BODIPY‐type systems to act as optical viscosity sensors by exploiting the restricted rotation of the peripheral substituents on this position. For example, in **14** ^[63]^ which encompasses styryl substitution on position 5 of the BODIPY core and mitochondrial targeting pyridinium cations, freely rotating trifluoromethyl group on the meso carbon leads to a bathochromic shift and negligible fluorescence. In viscous environments, the restricted flexibility of this motif enhances the fluorescence emission of the probe (φ_f_=0.03 and 0.26 for **15** in H_2_O and glycerol, respectively). It is noteworthy that the analogue system (**16**) bearing styryl substitution on both, positions 3 and 5 did not exhibit preferential accumulation within the mitochondria. Styryl substitutions on positions 3 and 5 are in fact ubiquitous in this class of materials, allowing for efficient conjugation between the core and the end groups on the styryl moieties. Along these lines, similar findings were reported for another set of materials (**17**–**26**) bearing this substitution on the 3 and/or 5 positions and heavy atoms on positions 2 and 6.^[65]^

Mono‐substituted **17**–**21** were observed to reduce cell viabilities at low micromolar concentrations and to not preferentially accumulate within the mitochondria. Symmetrical analogues exhibited lower cytotoxicity but preferential accumulation in subcellular organelles other than the mitochondria. Probe **27**, another asymmetrical system with pyridine and pyridinium groups linked through styryl substitutions onto the BODIPY core were reported.^[58]^ These together with a para‐azide phenyl on the meso position afforded a red shifted probe.

Piperidinyl based styryl BODIPYs have also been investigated.^[64]^ It was observed that both mono‐ (position 3) and di‐ (position 3 and 5) substituted systems (**28** and **29**, respectively) were characterized by mitochondrial accumulation whilst being neutral. It is of note that these architectures are characterized by significantly larger Stokes shifts (Δλ) when compared to other BODIPY systems (Δλ=2862.5 and 1368.7 cm^−1^ for **28** and **29** in acetonitrile, respectively), hence alleviating a major concern in the utilization of BODIPY chemistries for bioimaging applications. In this regard, the largest Stokes shift, Δλ=3006.2 cm^−1^ for a BODIPY system was reported for **30**, an architecture bearing pyridinium mitochondrial targeting unit on position 1 and no meso substitution.^[58]^ Similarly, increments in Stokes shifts were accessed for **31** and **32**, both bearing terminal triphenylphosphonium units.[^56^, ^77^] These systems indicate that greater Stokes shifts can also be accessed via larger peripheral substitutions on other than the meso position. Utilization of trifluoromethyl substitution on neutral BODIPY based architectures (**33** and **34**) resulted in subcellular accumulation within the mitochondria but also in the lysosomes.^[60]^ More recently, another example bearing styryl substitution on position 3 reported successful access to mitochondria by inclusion of an end triphenylphosphonium group on the meso substituent (**35**).^[81]^ It was observed that the absence of the mitochondrial targeting motif in the parent system led to accumulation in different organelles. Another probe (**36**), sharing some structural similarities with **35** was reported.^[76]^ It was observed that the location of the triazole and triphenylphosphonium units on position 3 and not 8 (meso) did not compromise the selective probe accumulation within the mitochondria.

These observations reinforce the known complexity of inducing mitochondrial selective accumulation by means of neutral systems.[^55^, ^75^] However, successful attempts on this regard have been reported exploiting BODIPY chemistries. An interesting set of structural analogues were reported (**37‐**‐**41**), where small substitutions were observed to dramatically dictate the accumulation in the mitochondria.^[61]^ In short, for systems bearing meso benzyl chloride substitution (**37** and **38**), mitochondrial accumulation was only observed when a formyl moiety is present on position 6 (**37**) and not on 5 (**38**), at the expense of a significant decrease in quantum efficiency (φ_f_=0.02 and 0.40 for **37** and **38** in DMSO, respectively). Interestingly, replacing the meso substitution with other aromatic groups (e. g., tolyl, mesityl) whilst maintaining the formyl moiety on position 5 of the core motif (**39**–**41**) also resulted in mitochondrial accumulation which indicates a synergistic effect. These substitutions were further observed to increase the quantum yield when compared to that of **37**, hence representing superior alternatives. This work also reported that H‐ or Me‐(meso) substituted systems were not observed to accumulate within the target organelle. The role of systematic substitution in a series of meso‐phenyl probes bearing methylpyridinium as mitochondrial targeting unit has also been evaluated.^[62]^ Importantly for design purposes, analogues to their successful probe (**42**) bearing either selenophene or bromine substitution on positions 2 and 6 were observed to be cytotoxic. There also have been other attempts at employing selenium in meso‐selenide neutral BODIPYs. However, results were inconclusive and could not confirm the localization of the probe in the target organelle (**4**, **5** and **6**).^[67]^

The use of charged mitochondrial targeting units other than delocalized lipophilic cations in BODIPYs has been explored.^[57]^ Interestingly, it was found that replacing ubiquitous triphenylphosphonium (**43**) for triethylammonium (**44**) afforded comparable selective accumulation while enhancing the quantum efficiency (φ_f_=0.49 and 0.69 for **43** and **44** in water, respectively). This can be partially ascribed to improved water solubility and lower probability of quenching from aggregation through newly available radiationless deactivation pathways. The use of triphenylsulfonium was also evaluated,^[80]^ with the generated probe (**45**) exhibiting selective mitochondrial accumulation. On the contrary, mitochondrial accumulation was attempted unsuccessfully by a neutral meso pyridine system (**46**) with aryl substitutions on positions 1/7 (thiophene) and 3/5 (phenyl) whilst its pyridinium analogue exhibited preferential localization in the target organelle (**46**).^[66]^ Progression from **46** to **47** also afforded a significant bathochromic shift to both absorption and fluorescence emission maxima (λ_abs/em_^max^=635/661 and 665/706 nm for **46** and **47** in DMSO, respectively) at the expense of diminishing the quantum efficiency (φ_f_=0.52 and 0.26 for **46** and **47** in DMSO, respectively).

## Coumarins

Coumarins denote a large family of naturally occurring materials that were first extracted from tonka beans in the second decade of the 19^th^ century.[^54^, ^86^] Since then, they have attracted an increasingly large surge of interest of the medicinal chemistry field on account of their biological activity and their role in the development of new drugs. Importantly, in the last decade the coumarin scaffold has been widely exploited in the development of systems for selective mitochondrial imaging. Synthetically, green chemistries have been shown to facilitate the realisation of coumarins with high yields.[^87^, ^88^] In such efforts, where the deep eutectic solvents act as both solvent and catalyst, methylene compounds and salicylaldehydes bearing desired substitution are employed under Knoevenagel conditions.^[87]^ Substituted salicylaldehydes can also be reacted with phenylacetic/cyanoacetic acids under Perkin conditions to yield desired coumarin‐based platforms.[^89^, ^90^] There are reports on accessing coumarin architectures from phenols and β‐ketoesters under Pechmann condensation conditions with sulfuric acid as catalyst.^[91]^ Lastly, the synthesis of this class of scaffolds has also been reported from ketones,[^92^, ^93^] such as the reaction of hydroxy acetophenone with diethyl carbonate following Claisen condensation conditions in the presence of sodium hydride.^[94]^

In relation to the photophysical properties of coumarin‐based systems, it should be noted that the core structure is characterized by absorption and fluorescence emission restricted to the UV spectral region. As a result, peripheral alterations are essential in accessing probes with optical behavior in the visible region. This also aids in increasing the intrinsically low absorption factor of coumarins, often leading to poor brightness. A ubiquitous alternative to improve such poor performance associated to these chemistries is the extension of the conjugation through fused rings on positions 5–6/6–7 (Figure 2).[^95^, ^96^, ^97^, ^98^] However, similarly to the case with other core motifs, we consider these to be out of the scope of this work. Peripheral modifications also lead to significant structural flexibility and conformational differences between relaxed excited and ground states, with associated large Stokes shifts. In some cases (**48** and **49**, Table 2), structural modifications result in rigid molecules which as a consequence are characterized by smaller Stokes shifts than those normally observed in coumarins. These probes employ ammonium^[99]^ and chromene^[100]^ mitochondrial targeting units, respectively. In this regard, reported probes exploiting coumarin chemistries for mitochondrial accumulation and imaging are not solely restricted to the use of ubiquitous triphenylphosphonium (**50**)^[101]^ and pyridinium delocalized lipophilic cations. Other charged moieties such as quinolinum, benzothiazolium, pyrrolidinium or indolium have been reported to successfully target this subcellular organelle.

**Table 2:** Chemical structure, absorption and fluorescent emission maxima (λ_abs/em_^max^/nm), Stokes shifts (Δλ/cm^−1^), fluorescence quantum yield (φ_f_) and solvent use for the characterization of reported coumarin‐based architectures.

Molecule	Chemical structure	λ_abs/em_^max^/nm	Δλ/cm^−1^	φ_f_	Solvent
**48^[a]^**	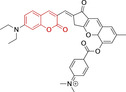	525/578	1746	–	DMSO/PBS
**49**	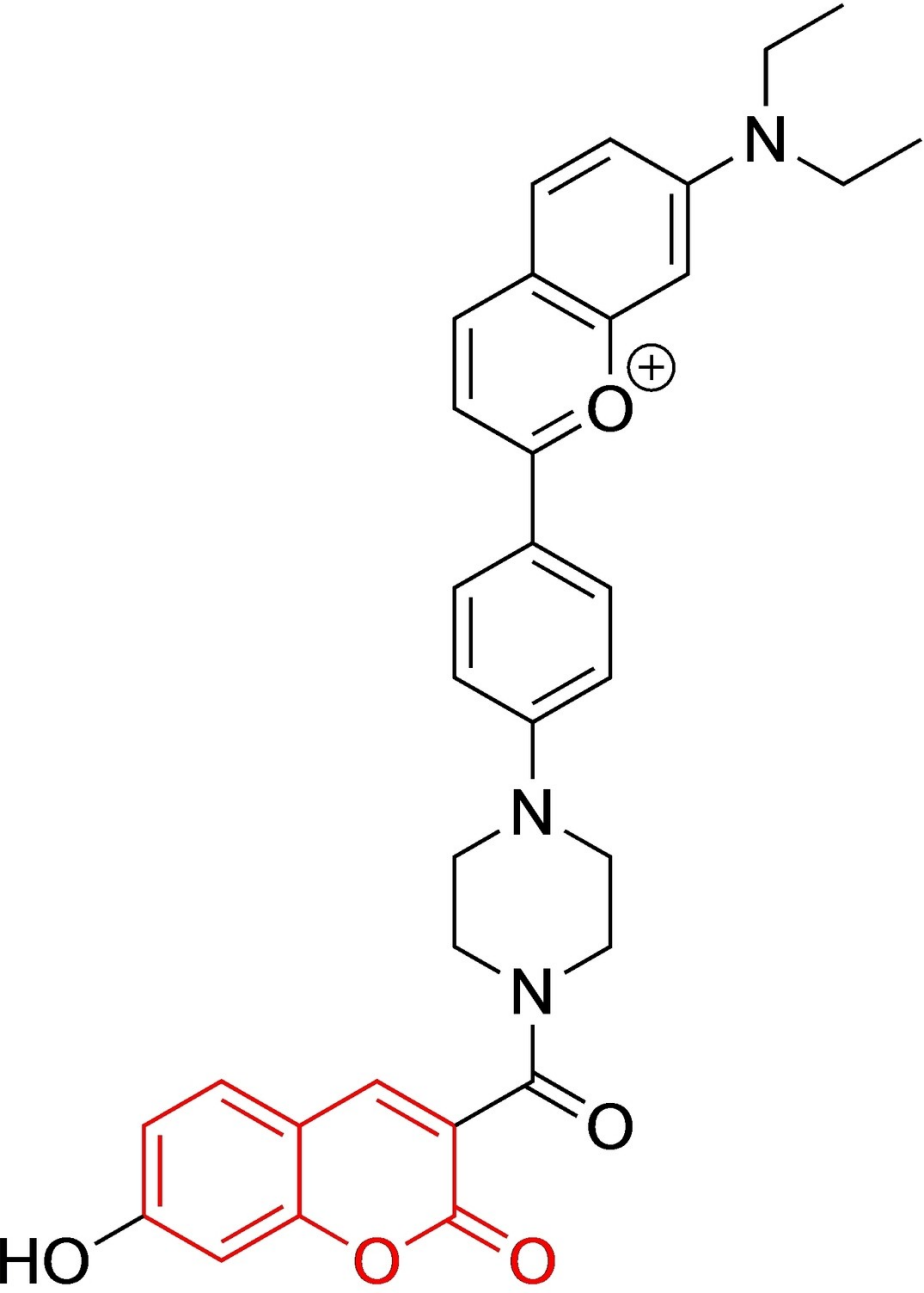	570/635	1795	0.05	PBS
**50**	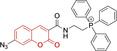	370/450	4804	0.05	Aqueous buffer
**51**	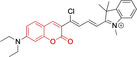	595/769	3802	0.20	DMSO
**52**	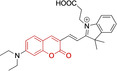	577/655	2063	–	PBS
**53**	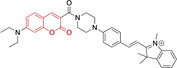	418/587	6887	<0.01	PBS
**54**	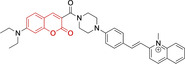	425/478	2608	0.15	DMSO:PBS (4 : 6)
**55**	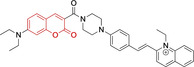	423/475	2588	0.01	Ethanol/PBS
**56**	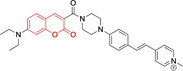	417/486	3404	–	Ethanol/PBS (5 : 95)
**57**	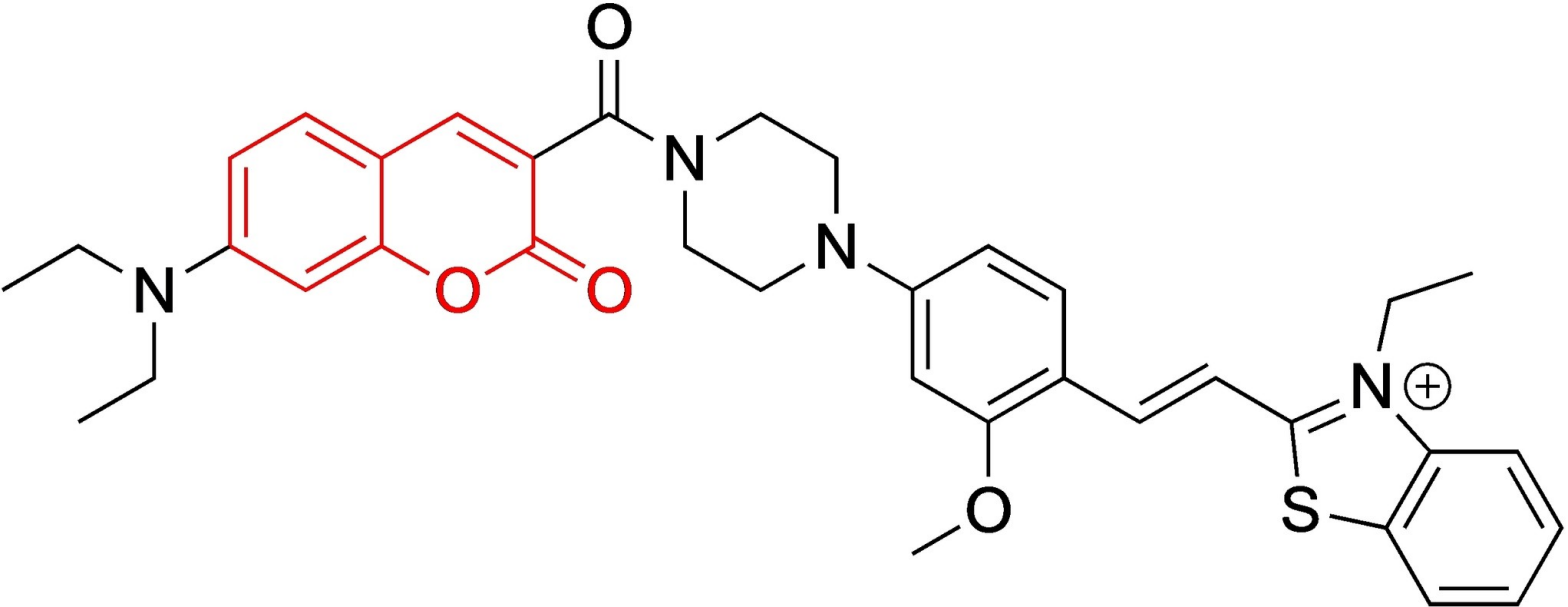	418/456	1993	0.03	PBS (30 % DMF)
**58**	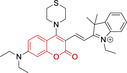	552/650	2731	–	PBS (30 % DMSO)
**59^[a]^**		440/630	6854	–	‐
**60**	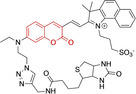	582/652	1844	–	PBS (30 % DMF)
	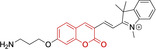				
**61**	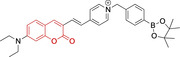	500/640	4375	–	Acetonitrile/PBS (1 : 9)
**62**	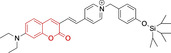	490/639	4758	–	DMF‐PBS (7 : 3)
**63**	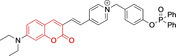	503/643	4328	–	PBS (50 % DMF)
**64**	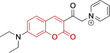	460/494	1496	<0.01	PBS
**65**	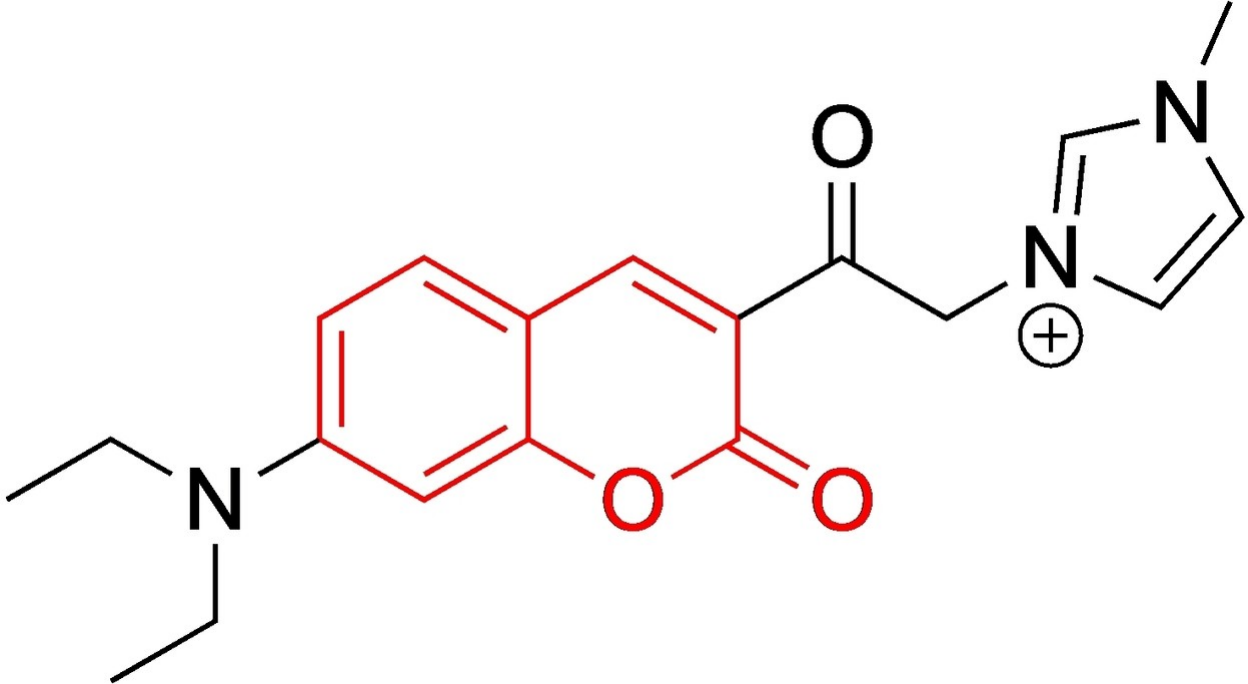	456/501	1969	<0.01	PBS
**66^[a]^**	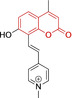	371‐542/‐	–	–	DMSO/PBS (1 : 5)
**67**	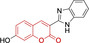	420/480	2976	0.65	Buffer
**68^[a]^**	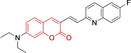	440/556	4741	0.38	–
**69^[a]^**	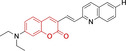	443/551	4424	0.31	–
**70^[a]^**	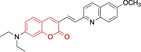	441/548	4427	0.24	–

^[a]^ Absorption and fluorescence emission maxima approximated from spectra.

Diethylamino substitution on position 7 of the coumarin core motif is present in the majority of reported systems. It has been observed (**51**) that impairment of the rotation of this functionality in viscous solvents results in a φ_f_ increase.[^102^, ^103^] Similarly to this probe, others systems exploited indolium cations to target mitochondria. Addition of an acid group on the indolium nitrogen in **52** results in a significant hypsochromic shift in the emission spectra (λ_em_^max^=769 and 655 nm for **51** and **52** in DMSO and PBS, respectively).^[104]^ Nonetheless, it is observed that conjugation of the indolium moiety to the coumarin core via styryl substitution denotes an efficient approach to induce bathochromic shifts in these systems. Along these lines, lengthening the linker between this mitochondrial targeting unit and the coumarin core in **53** was reported to yield significantly blue‐shifted absorption and emission spectra.^[105]^ Progression from **53** to **54** by substitution of the indolium moiety for quinolinium was observed to increase the quantum efficiency, albeit determined in different solvent systems.^[106]^ In this regard, it was then reported that methyl (**54**) vs. ethyl (**55**) substitution on the quinolinium nitrogen atom was responsible for a significant decrease in the quantum efficiency (φ_f_=0.15 and 0.01 for **54** and **55** in ethanol:PBS and DMSO:PBS, respectively).^[107]^ This observation could be in fact attributed to known large uncertainties in the relative determination of this photophysical parameter and not to arise from structural contributions.^[108]^ Given the length of the linker, it was demonstrated in **56** and **57** that whilst substitution of quinolinum in **55** for pyridinium (**56**) and benzothiazolium (**57**) ensured mitochondrial accumulation, it bears little effect on the photophysical properties.[^99^, ^109^]

Selective mitochondrial targeting in coumarin systems has also been facilitated by means of pyrrolidinium cation. Thiomorpholine substitution on position 4 in the case of **58** ^[111]^ was observed to result in a significant bathochromic shift in the absorption spectra and diminish in the Stokes shifts when compared to its structural analogue **59** ^[112]^ (λ_abs/em_^max^=552/650 and 440/630 nm for **58** and **59**, respectively). It is noteworthy that the significantly larger peripheral alternations performed in **59** when compared to **57** only resulted in bathochromic changes in the absorption spectrum (λ_abs/em_^max^=552/650 and 582/652 nm for **58** and **60**, respectively).^[113]^ The diethylamino substitution on position 7 has also been exploited, with pyridinium cations. It was observed that para‐substitution on the benzyl groups of the pyridinium nitrogens induced negligible differences in both the absorption and fluorescence emission spectra (λ_abs/em_^max^=500/640, 490/639 and 503/643 nm for **61, 62** and **63**, respectively). However, the rationale behind the choice of terminal boronate (**61**),^[114]^ isopropylsilane (**62**)^[115]^ and diphenylphosphine oxide (**63**) groups was the bifunctionality of the probes. Nonetheless, significant reduction of the conjugation of position 3 of the coumarin core on progression from these systems to **64** and **65** (imidazolium targeting cation) results in bathochromic spectral shifts and characteristic small Stokes shifts of coumarins.^[116]^ In turn, a large Stokes shift was observed for a probe (**66**) which unlike any other coumarin reported architecture, bear substitution on position 8 and not 3.^[117]^ Unfortunately, the photophysical properties of this pyridinium‐containing system were not investigated in detail to allow for the identification of structure property relationships.

Lastly, a number of neutral coumarin‐based platforms have been reported with successful mitochondrial accumulation. Compound **67**, as **66** was designed with the less frequently used hydroxyl substitution on position 7 of the core motif as opposed to the ubiquitous diethylamino.^[118]^ This along with the benzimidazole moiety on position 3 facilitated the largest reported quantum efficiency for a coumarin‐based architecture with preferential mitochondrial accumulation (φ_f_=0.65 for **67** in buffered solution). Probes **68–70** were designed with quinoline and diethylamino substitutions on positions 3 and 7, respectively.^[119]^ Preferential accumulation on these were associated to functional groups on the quinoline moieties, with best results observed for the fluorine‐substituted analogue. In turn, replacing this for H‐ or methoxy groups was observed to lead to poorer accumulation, hence indicating a potentially important role of fluorine in strengthening non‐covalent interactions.[^120^, ^121^]

## Cyanines

Alongside diketopyrrolopyrroles (see below), cyanines have been extensively exploited in the pigment industry. In the bioimaging field, they denote one of the most commonly used motifs, with cyanine‐based materials being known for exhibiting high brightness on account of their large absorption factors – despite often observed low fluorescence quantum yields ‐ and narrow absorption bands that extend into the NIR region.[^122^, ^123^] Structurally, cyanines are characterized by polymethine chains, terminated with nitrogen‐bearing groups at either end. Whilst extension of the conjugation of the polymethine chains affords bathochromically shifted architectures, these become increasingly unstable as a result of this structural modification. In this regard, tri‐, penta‐ and heptamethine chains are commonly utilized in cyanine‐based molecular systems. However, heptamethine certainly represent the most widely exploited motif, such as indocyanine green which is FDA approved for use in medical diagnostics.^[124]^ This probe belongs to a class of cyanines whereby the nitrogen atoms at either end of the polymethine chain are part of heterocyclic moieties. Synthetically, these can be accessed via direct non‐catalyzed condensation on heating of N‐alkyl substituted quaternary salts derived from 2,3,3‐trimethylindole or 2,3,3‐trimethylbenzindole and 2‐chloro‐1‐formyl‐3‐(hydroxy methylene)cyclohex‐1‐ene.[^122^, ^123^] Although cyanines platforms where only one (hemicyanines) or none (streptocyanines) of the nitrogen atoms are part of heterocyclic systems also conform to this class of materials, such design strategies are scarce in the available literature for bioimaging agents, particularly in the case of the latter. Hemicyanines, can be synthetized by condensation of a methyl quaternary salt with substituted benzaldehyde in the presence of a suitable base.^[123]^

When charged, the terminal nitrogen‐bearing moieties act as mitochondrial targeting units. Thus, the exploitation of delocalized lipophilic cations as peripheral substitutions in cyanines is lower when compared to other scaffolds reviewed in this work. In light of the technological success of indocyanine green, cyanine‐based materials bearing heptamethine chain and terminal indole/indolium motifs have been widely exploited for bioimaging applications. Substitutions on the central position of the polymethine chain, often referred to as the meso position, are ubiquitous. Incorporation of an anthracene motif in the meso position in **71** (Table 3) was observed to lead to specific mitochondrial accumulation despite the often‐reported concerns of inducing target organelle accumulation by means of a single indolium moiety.^[125]^ Similar observations were reported for **72**, a pentamethine system bearing identical terminal substitutions and hydroxy on the meso position. However, the former was bathochromically shifted as a result of the extended conjugation from the anthracene moiety.^[126]^ Interestingly, replacement of anthracene for bromine in **73** achieved comparable spectral maxima despite the shorter polymethine chain (λ_abs/em_^max^=650/670 and 634/654 nm for **71** and **73** in DCM and PBS, respectively).^[127]^ For continuous irradiation purposes, photostability of the probes denotes a very highly desirable characteristic. In this regard, the integrity of heptamethine chains can be enhanced by the presence of a cyclohexene moiety in the centre of the chain. In particular, the use of chlorine bearing cyclohexenes is widely exploited in cyanines, such as **74** which also exhibits a significant bathochromic shift as a result of the substitution.^[128]^ This can be attributed to the enhanced planarity across the central chain. Compound **75** and **76** both bear central cyclohexene and morpholine substitution linked to the indolium nitrogen atom through a long alkyl chain. In line with concerns associated to single indolium targeting units, both probes exhibited poor mitochondrial accumulation.^[129]^ While **75**, encompassing chlorine substitution on the central cyclohexene was observed to accumulate in the lysosomes, replacing it for piperazine allowed for mitochondrial accumulation, albeit not selective nor preferential. Their analogues, **77**,^[130]^ **78** ^[131]^ and **79** ^[132]^ bearing substitution on both indole and indolium nitrogen atoms exhibited superior mitochondrial accumulation. Due to the terminal nature of their substitutions on the alkyl chains of the nitrogen atoms, these were characterized by comparable photophysical properties, even in the case of thymine substituted **78**. In turn, substitutions on the central chlorocyclohexene moieties in **80** and **81** were observed to only afford preferential and not specific mitochondrial accumulation.^[133]^ This is of particular note in the case of **81**, where the use of a piperidinium analogue as the central moiety did not facilitate targeted accumulation.

**Table 3:** Chemical structure, absorption and fluorescent emission maxima (λ_abs/em_^max^/nm), Stokes shifts (Δλ/cm^−1^), fluorescence quantum yield (φ_f_) and solvent use for the characterization of reported cyanine‐based architectures.

Molecule	Chemical structure	λ_abs/em_^max^/nm	Δλ/cm^−1^	φ_f_	Solvent
**71**	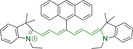	650/670	459	0.30	DCM
**72**	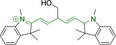	540/560	661	0.03	PBS (φf in water)
**73**	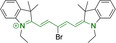	634/654	482	0.03	PBS
**74^[a]^**	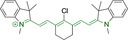	780/795	241	–	FBS
**75**	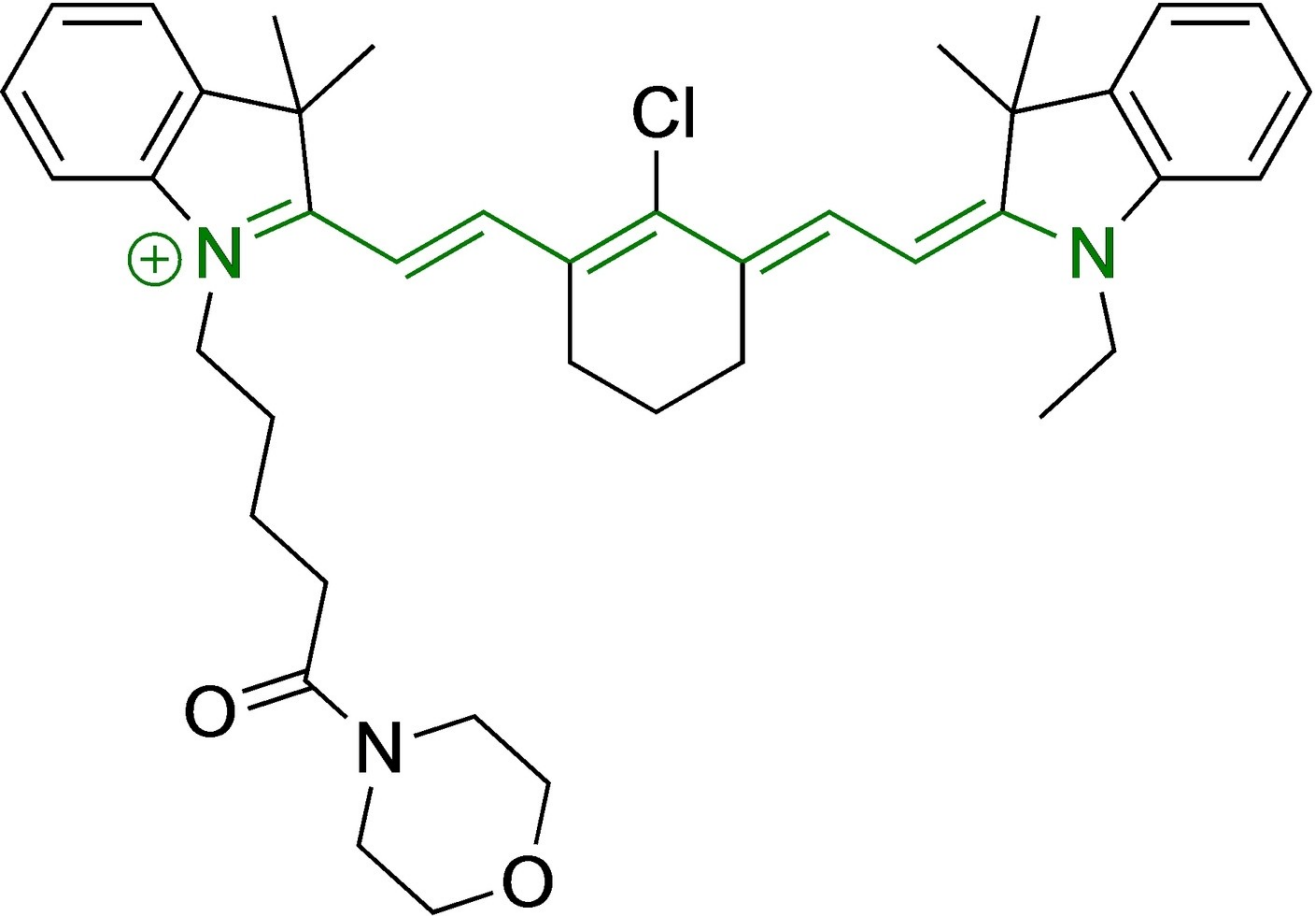	794/828	517	0.18	DMSO
**76**	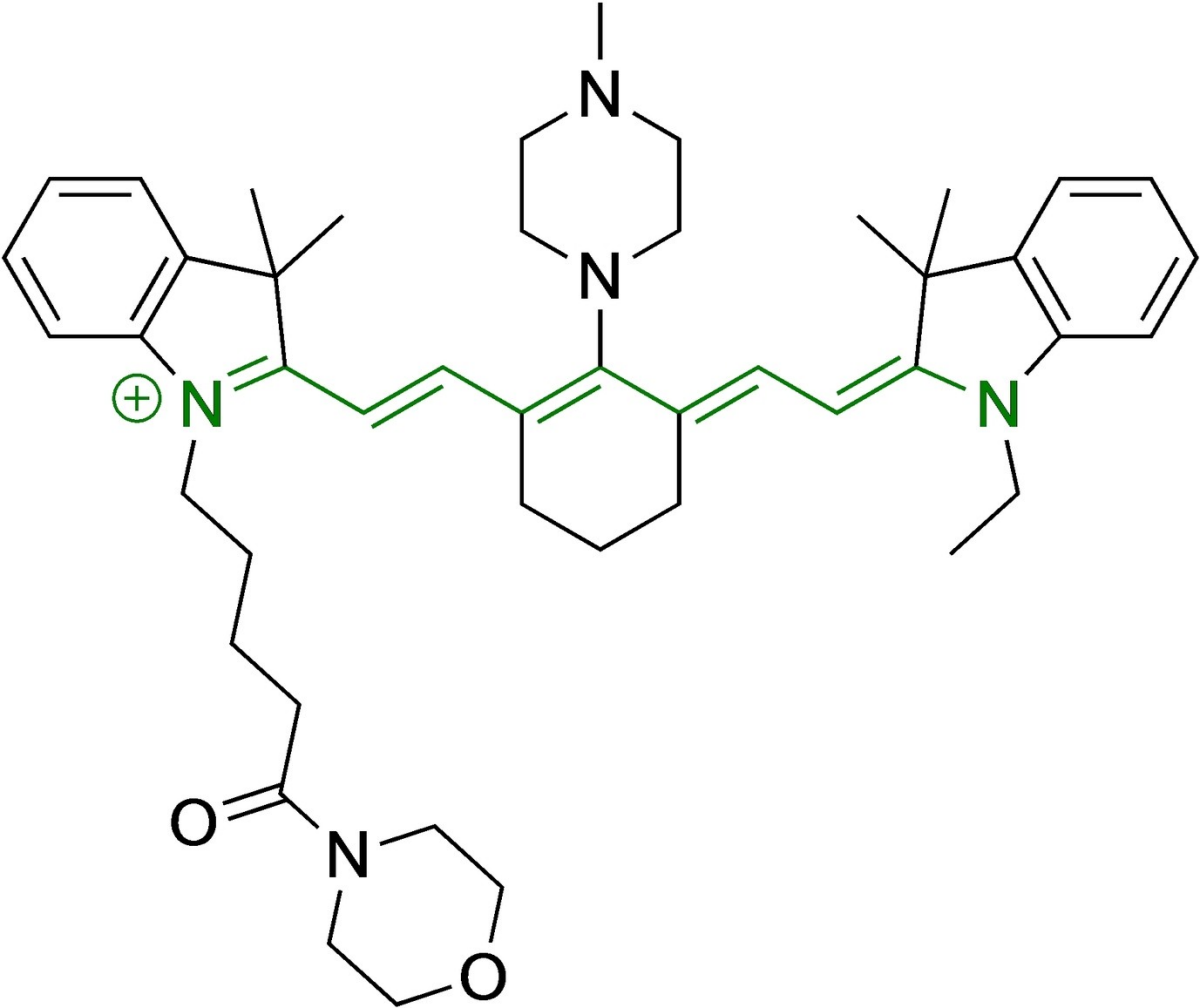	704/812	1889	0.21	DMSO
**77**	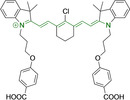	781/799	288	0.04	MeOH
**78^[a]^**	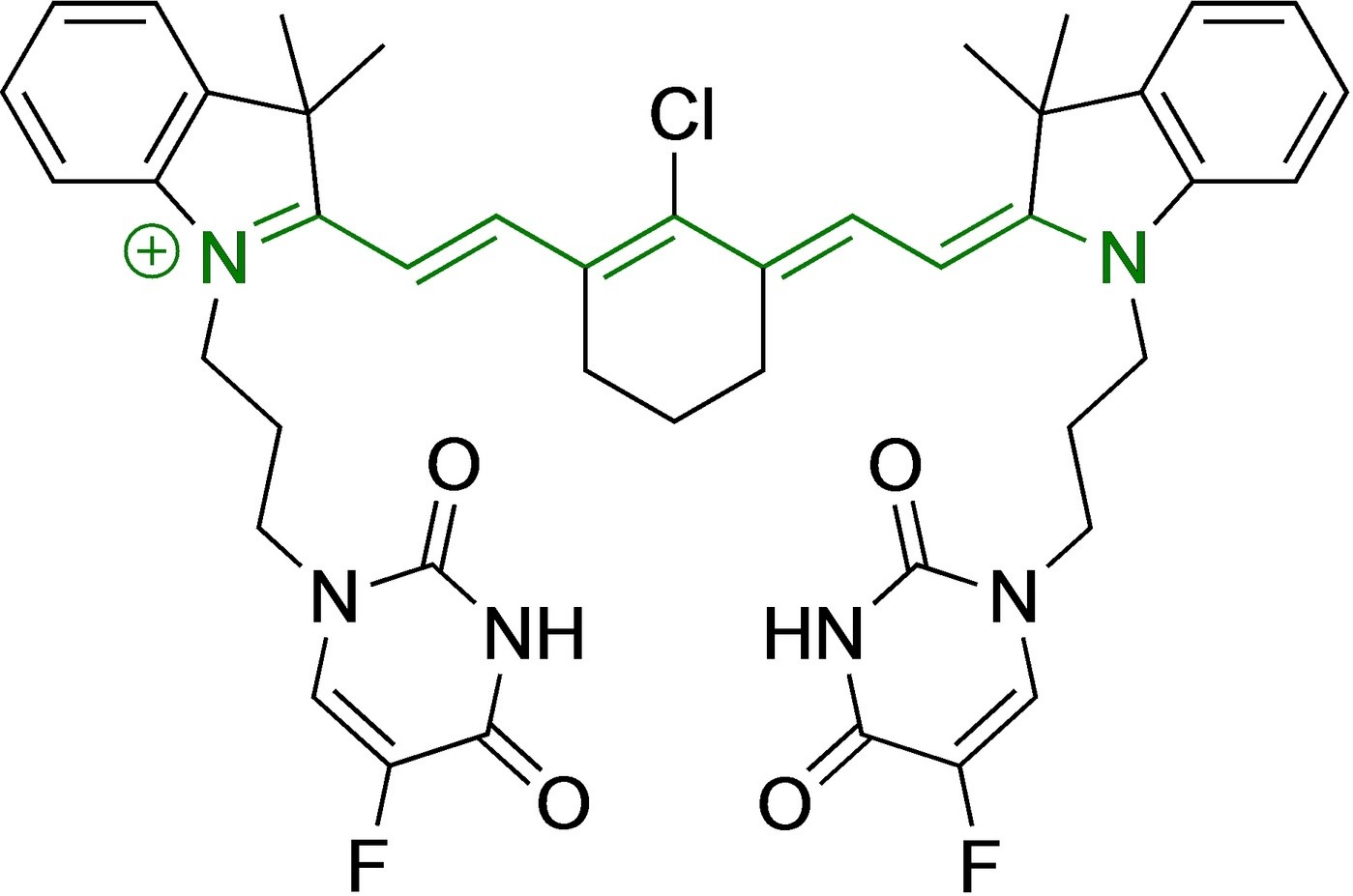	775/800	403	–	Sodium citrate buffer
**79**	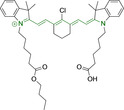	782/804	349	0.08	Methanol
**80**	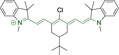	768/793	410	0.03	HBSS buffer (1 % DMSO)
**81**	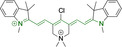	739/758	339	0.04	HBSS buffer (1 % DMSO)
**82**	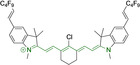	804/816	182	0.08	Methanol
**83**	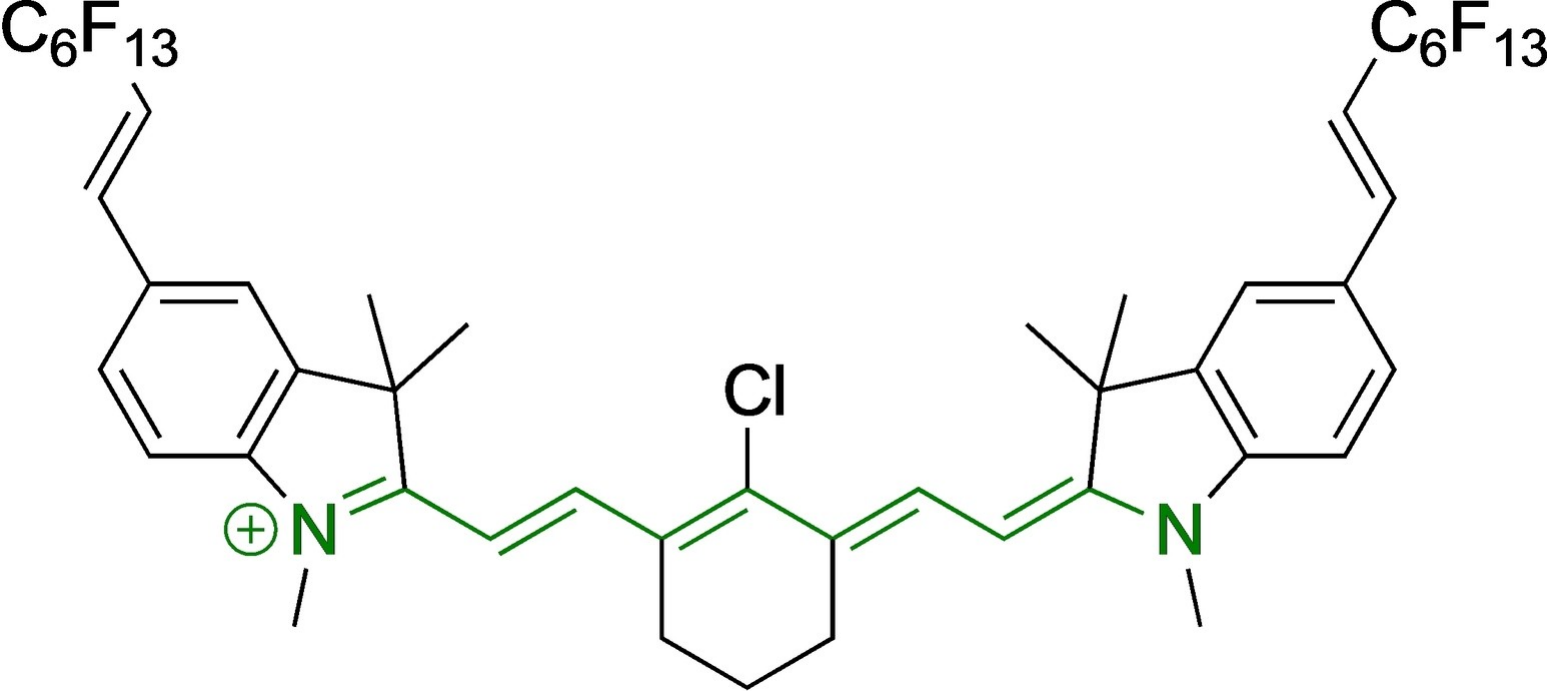	803/820	258	0.08	Methanol
**84**	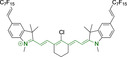	805/817	182	0.08	Methanol
**85**	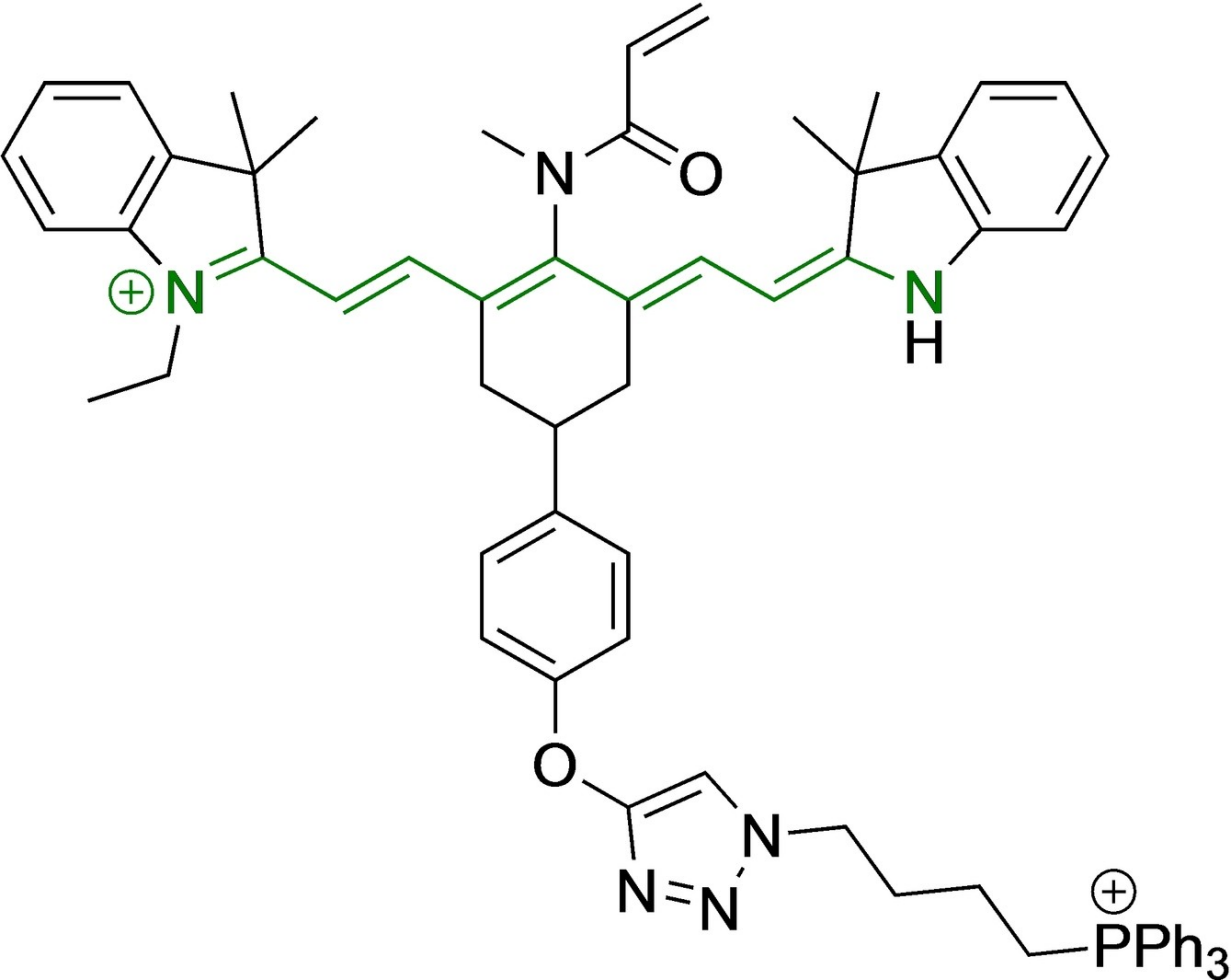	784/825	633	–	HEPES buffer
**86**	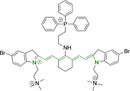	627/746	2544	–	Methanol
**87**	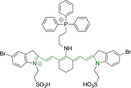	618/748	2812	–	Methanol
**88**	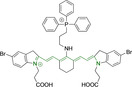	630/750	2539	–	Methanol
**89**	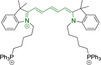	644/663	444	0.30	Water (φf in DMSO)
**90**	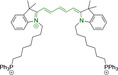	645/663	420	0.24	Water (φf in DMSO)
**91**	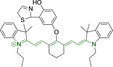	770/795	408	0.17	Acetonitrile/PBS (1 : 1)
**92**	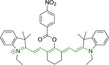	780/785	81	–	HEPES buffer
**93**	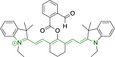	765/780	251	–	HEPES buffer (0.5 % acetonitrile)
**94**	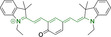	575/700	3105	–	PBS
**95**	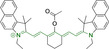	800/825	378	–	HEPES buffer
**96**	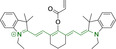	759/794	580	0.08	HEPES buffer
**97**	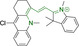	592/660	1740	0.01	PBS 0.1 % DMSO)
**98**	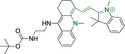	509/640	4021	0.18	PBS (0.1 % DMSO)
**99**	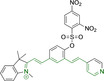	380/604	9759	–	PBS (10 % DMSO)
**100**	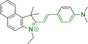	550/611	1815	–	PBS
**101**	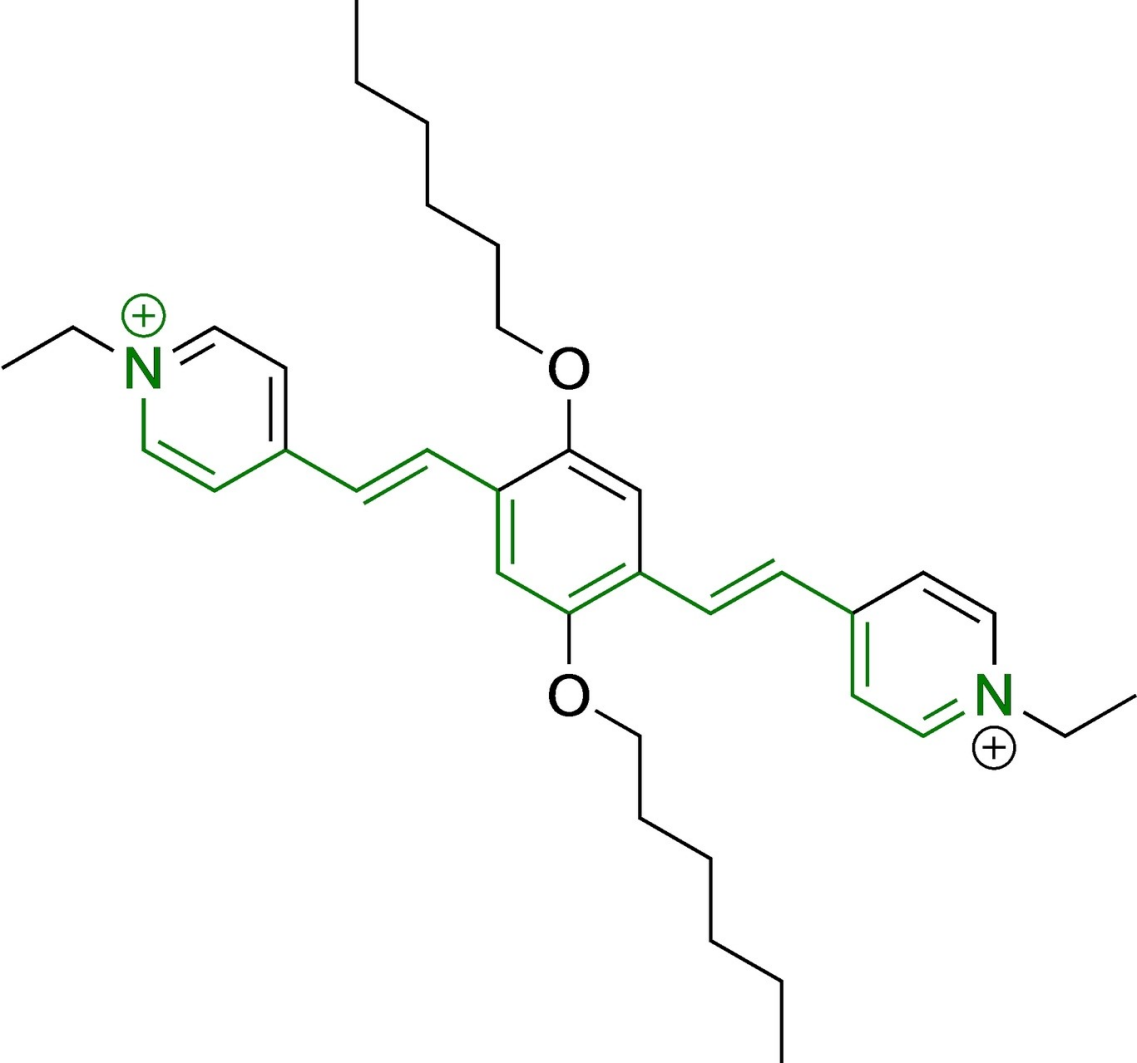	440/608	6279	0.06	Water
**102**	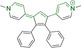	520/617	3023	<0.01	chloroform
**103**	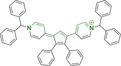	533/606	2260	<0.01	chloroform
**104**	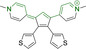	520/589	2252	<0.01	chloroform
**105**	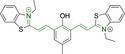	382/770	13191	0.01	Water
**106**	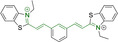	380/460	4576	‐	Water
**107**	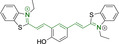	405/710	10606	0.03	Water
**108**	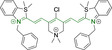	754/774	342	0.08	HBSS buffer (1 % DMSO)
**109**	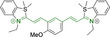	406/574	7208	0.04	Water
**110**	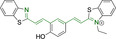	424/620	7455	<0.01	Water
**111**	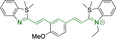	408/602	7898	<0.01	Water
**112**	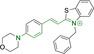	454/597	5276	<0.01	Water

A different approach was presented by probes **82–84**, bearing isosteric fluorine substitutions as peripheral alterations on the indole/indolium moieties.^[124]^ Their structural differences were observed to influence their accumulation but not their photophysical behavior. As such, the best mitochondrial accumulation was reported for **83**. In an attempt to optimize selective mitochondrial targeting, a number of probes have exploited the use of additional delocalized lipophilic cations, such as triphenylphosphonium in **85**, in addition to cyanine indolium groups.^[134]^ Similarly, **86–88** exploited this delocalized lipophilic cation linked to a secondary amine on the meso position of their heptamethine chain.^[135]^ Their large quantum yields when compared to those commonly observed in cyanine‐based systems (e. g., Δλ=339.19 and 2544.14 cm^−1^ for **81** and **86** in Hank's Balanced Salt Solution (HBSS) buffer and methanol, respectively) can be attributed to the flexibility of this peripheral substitution. Probe **86** further incorporates trimethylammonium groups linked to the indole/indolium groups, conferring this probe with best mitochondrial accumulation when compared to its analogues, **87** and **88**. Triphenylphosphonium units were also utilized in indole/indolium probes, **89** and **90** where the lack of central cyclohexene can be ascribed to the use of penta‐ and not heptamethine chains.^[136]^ Mitochondrial targeting units were incorporated in terminal positions of alkyl chains on the nitrogen atoms. Both probes were observed to accumulate within the target organelle and the longer alkyl chain in **90** did not result in any significant changes to the photophysical properties or their selective accumulation. Preferential targeting was possible for a number of probes bearing single indolium motifs, such as in **91**, bearing ether substitution on to the meso position.^[137]^ Progression from this to acid derivative substitution in **92** ^[138]^ and **93** ^[139]^ was observed to result in small changes to the photophysical parameters, including narrow Stokes shifts.^[138]^ In turn, a significant hypsochromic shit and larger Stokes shifts can be observed in **94**.

The presence of the ketone in the central cyclohexadiene was observed to limit selective accumulation within the mitochondria with some accumulation also in the lysosomes and endoplasmic reticulum.^[140]^ Bathochromic shifts can also be accessed by substitutions on the terminal indole/indolium units. Addition of a fused phenyl group in **95** ^[141]^ was observed to lead to a ca. 40 nm shift when compared to its indole/indolium counterpart **96** (λ_abs/em_^max^=800/825 and 759/794 nm for **95** and **96** in 4‐(2‐hydroxyethyl)‐1‐piperazineethanesulfonic acid (HEPES) buffer, respectively).^[142]^ Mitochondrial targeting was also approached by platforms bearing a single indolium moiety. Interestingly, successful accumulation was reported for **97** and **98**,^[143]^ whilst **99** was observed to partially accumulate within the nuclei as well.^[144]^ Progression from **97** to **98**, where the reactive chlorine is replaced for an aminocarbamate, results in a hypsochromic shift and increase in the Stokes shift (λ_abs/em_^max^=592/660 and 509/640 nm for **97** and **98** in phosphate buffer, respectively). This can be ascribed to greater structural flexibility in the latter. The counterintuitive increase in the quantum efficiency as a result of this substitution can however be partially attributed to the observed spectral shift and diminish of the optical band gap. As previously discussed, the use of hemicyanines is scarce in the literature for bioimaging probes. Nonetheless, the terminal neutral dimethylamino moiety in indolium‐bearing **100** was used to monitor mitochondrial viscosity changes, albeit characterized by significantly hypsochromically shifted spectra as a result of the diminish in the conjugation through its molecular backbone.^[145]^

Alternative mitochondrial targeting motifs have also been exploited in cyanine systems, such as pyridinium moieties in **101**.^[146]^ Compound **102–104** were also observed to selectively accumulate in the mitochondria despite bearing single pyridinium units.^[147]^ These bear identical backbone, with a central pentadiene unit in their heptamethine chains with pendant aryl groups. Substitution of phenyl for thiophene on going from **102** to **104** resulted in a hypsochromic shift in the emission spectra and smaller Stokes shift as a result. In turn, it was of interest to see that addition of aryl groups onto the pyridine/pyridinium nitrogen atoms afforded negligible spectral shifts (λ_abs/em_^max^=520/617, 533/606 and 520/589 nm for **102, 103** and **104** in chloroform, respectively).

A successful alternative is the use of benzothiazolium motifs on both ends of the polymethine chains, such as in **105** and **106**.^[148]^ The large Stokes shift in **105** can be associated to the keto‐enol tautomerism in this system, absent in **106** due to the lack of hydroxyl substitution on the meso position (λ_abs/em_^max^=382/770 and 380/460 nm for **105** and **106** in water, respectively). Similarly, **107** is also characterized by large Stokes shift on account of such tautomerism. However, the different position of the hydroxyl group on the central benzene moiety diminished the extent of the bathochromic shift.^[130]^ Similarly to the case described for **80** and **81**, **108** was observed to only preferentially accumulate in the mitochondria despite the additional central pyridinium targeting motif.^[133]^ The hypsochromic shift observed in **107** was replicated by **109**, bearing methoxy instead of hydroxy substitution on the central moiety. Targeted mitochondrial accumulation of **107** and **109** was observed to be impaired by substituting one of the benzothiazolium moieties for neutral benzothiazole. In this regard, their analogues, **110** ^[130]^ and **111** ^[150]^ respectively, were observed to target the lysosomes. Mitochondrial accumulation in architectures bearing a single benzothiazolium was further evaluated in a pentamethine architecture where the second nitrogen atom is incorporated into a morpholine motif (**112**).^[151]^ However, this was observed to also accumulate in the lysosomes.

## Diketopyrrolopyrroles

2,5‐Dihydropyrrolo[4,3‐c]pyrrolo‐1,4‐diones (Figure 2), commonly known as diketopyrrolopyrroles (DPPs) have been widely used in the pigment industry as high performance pigments on account of their low solubility as a result of strong intramolecular H‐bonding interactions as well as high brightness and photostability.^[152]^ More recently, disruption of these strong interactions via N‐substitutions have led to large exploitation of the electron deficient π‐conjugated bicyclic dilactam core motif of DPPs in optoelectronic applications, where they are often characterized by their strong fluorescence and tunable properties through substitutions on positions 2/5 and 3/6 (Figure 2). Despite these observations, DPP‐based materials are still arguably underexploited in the bioimaging field when compared to other scaffolds reviewed in this work. DPPs were first obtained as a by‐product of the synthesis of an unsaturated β‐lactam under Reformatsky conditions.^[153]^ Due to a number of limitations, most symmetric (identical substituents on the 3 and 6 positions, Figure 2) DPP pigments are nowadays synthesized following a modified method, namely the succinic method where an aromatic nitrile is reacted with a dialkyl succinate in the presence of a metal alkoxide and a tertiary alcohol.^[52]^ Whilst symmetric DPPs are widely used in other applications, these are only appropriate for biological uses when bearing peripheral substitutions which induce water solubility. Alternatively, highly polar systems can be designed to ensure solubility in a vehicle solvent (i. e., used for solubility purposes) such as DMSO. Ideally, DMSO concentrations in cell media should be kept below 0.5 % v/v (and never above 1 %) to avoid a detrimental impact on cell viabilities.

DPPs with increased polarity are often afforded through asymmetric substitutions through the long molecular axis (i. e., the one running through positions 3/6 and the centre of the transannular bond).^[35]^ Unfortunately, the aforementioned succinic method is not appropriate for the synthesis of DPP pigments bearing different substitution at the 3 and 6 positions of the core motif. In most cases, this is achieved by reacting a β‐ketoester with bromoacetic acid under basic conditions and subsequent cyclation in the presence of ammonium acetate. The generated lactam is then reacted with a nitrile in the presence of an alkoxide to give the asymmetric DPP pigment. Subsequently, and irrespective of their molecular symmetry, progression from pigment to dye (i. e., N‐substituted) analogue is often afforded via alkylation under basic conditions.^[52]^

Whilst careful choice of peripheral substituents on positions 2 and 5 can significantly influence the photophysical properties in such materials, these are often overlooked and used solely to induce solubility. Along these lines, enhancing the electron density within the electron deficient DPP core motif by means of electron donating substituents on these positions is associated with observed bathochromic spectral shifts.^[52]^ However, most efforts in the design of novel molecules are devoted to structural alterations on positions 3 and 6 with long alkyl chains on the lactam nitrogens for solubility purposes.[^154^, ^155^, ^156^, ^157^, ^158^, ^159^] In this regard, it is nowadays often observed the selection of furan and thiophene as the core rings in detriment of original phenyl motifs.[^154^, ^155^] The main reasoning behind this choice is the observed bathochromic shifts associated to the increased planarity throughout the conjugated axis in these systems when the former are selected. This was nicely evaluated for phenyl (**113**), thiophene (**114**) and furan (**115**) bearing DPPs (λ_abs/em_^max^=495/576, 603/635 and 591/612 nm for **113**, **114** and **115** in DMSO, respectively, see, Table 4).^[158]^ Such core alterations were also consistent with hyperchromic (increase in absorption factor) spectral shifts (ϵ=2.60, 3.46 and 5.57×10^4^ M^−1^ cm^−1^ for **113**, **114** and **115** in DMSO, respectively). Yet, as a consequence of such strategies, these systems which make use of imidazolium targeting units, are characterized by a decrease in the Stokes shift on progression from **113** to **114** and **115**, which is detrimental for bioimaging applications due to greater probability of self‐absorption. This can be readily ascribed to their greater rigidity, lower structural re‐arrangements when promoting an electron and thus greater structural similarities on comparing relaxed ground and excited state geometries. It should also be noted that expansion of the conjugation along the long molecular axis beyond the core rings also results in bathochromic shifts, albeit not as significant due to out‐of‐plane motions. For instance, addition of a phenothiazine moiety to one of the thiophene core rings in asymmetric probe **116** resulted in both absorption (λ_max_=585 nm) and fluorescence emission (λ_max_=615 nm) maxima in line with those reported for non‐phenothiazine analogues.[^52^, ^155^] On the other hand, peripheral alternations throughout the long molecular axis are can also result in changes to absorption factors due to dipole moments for HOMO–LUMO transitions broadly coinciding with this molecular axis.

**Table 4:** Chemical structure, absorption and fluorescent emission maxima (λ_abs/em_^max^/nm), Stokes shifts (Δλ/cm^−1^), fluorescence quantum yield (φ_f_) and solvent use for the characterization of reported diketopyrrolopyrrole‐based architectures.

Molecule	Chemical structure	λ_abs/em_^max^/nm	Δλ/cm^−1^	φ_f_	Solvent
**113**	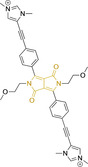	495/576	2840	0.89	DMSO
**114**	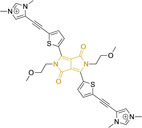	603/635	835	0.49	DMSO
**115**	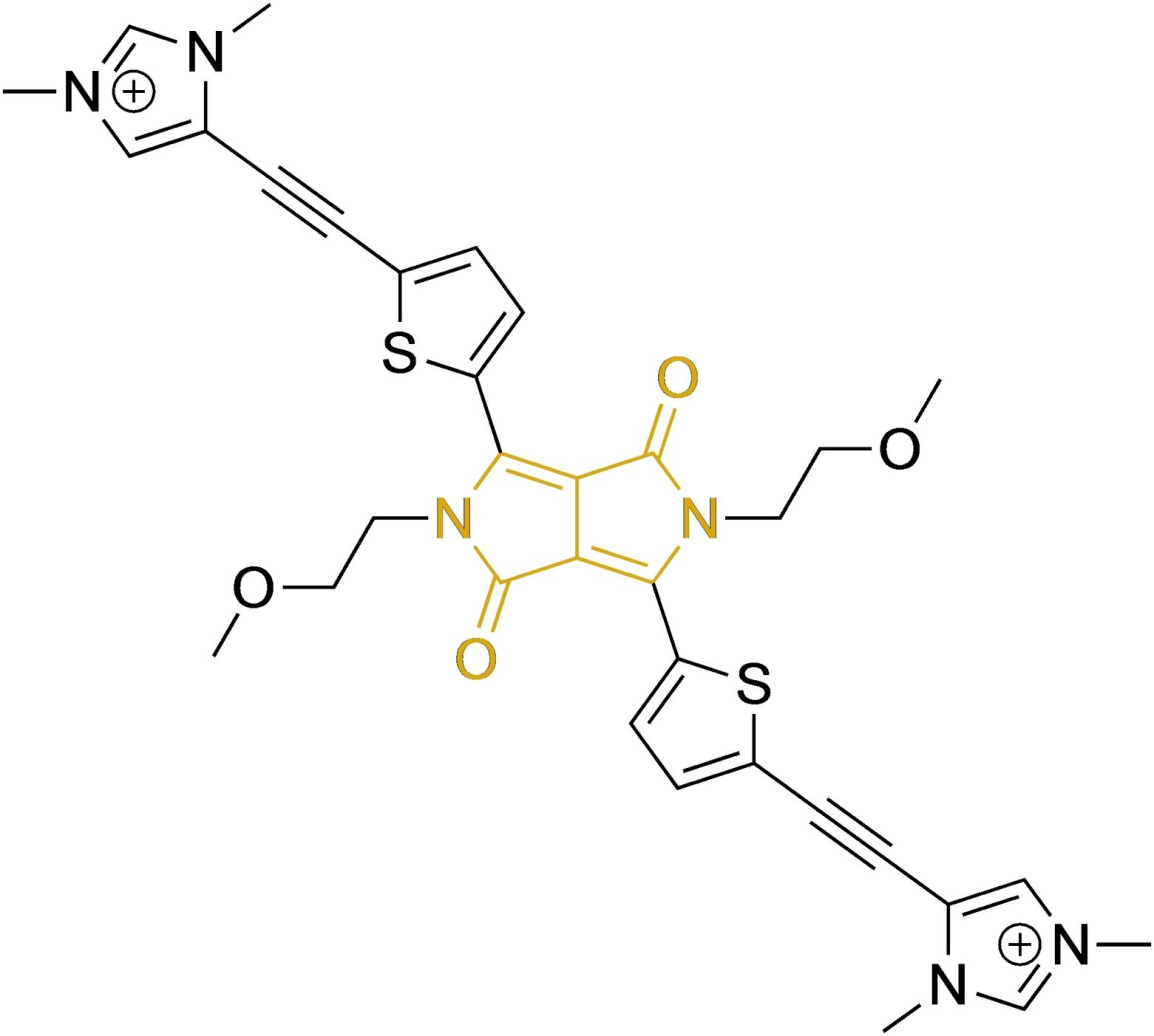	591/612	580	0.62	DMSO
**116**	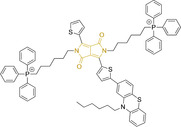	585/615	833	–	PBS
**117**	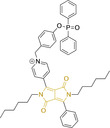	520/652	3893	–	Ethanol/PBS (3 : 7)
**118**	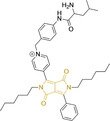	520/651	3869	–	Ethanol/PBS (2 : 8)
**119**	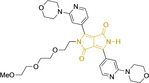	470/518	1971	0.01	Acetonitrile
**120**	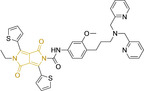	547/564	551	0.01	DMSO
**121**	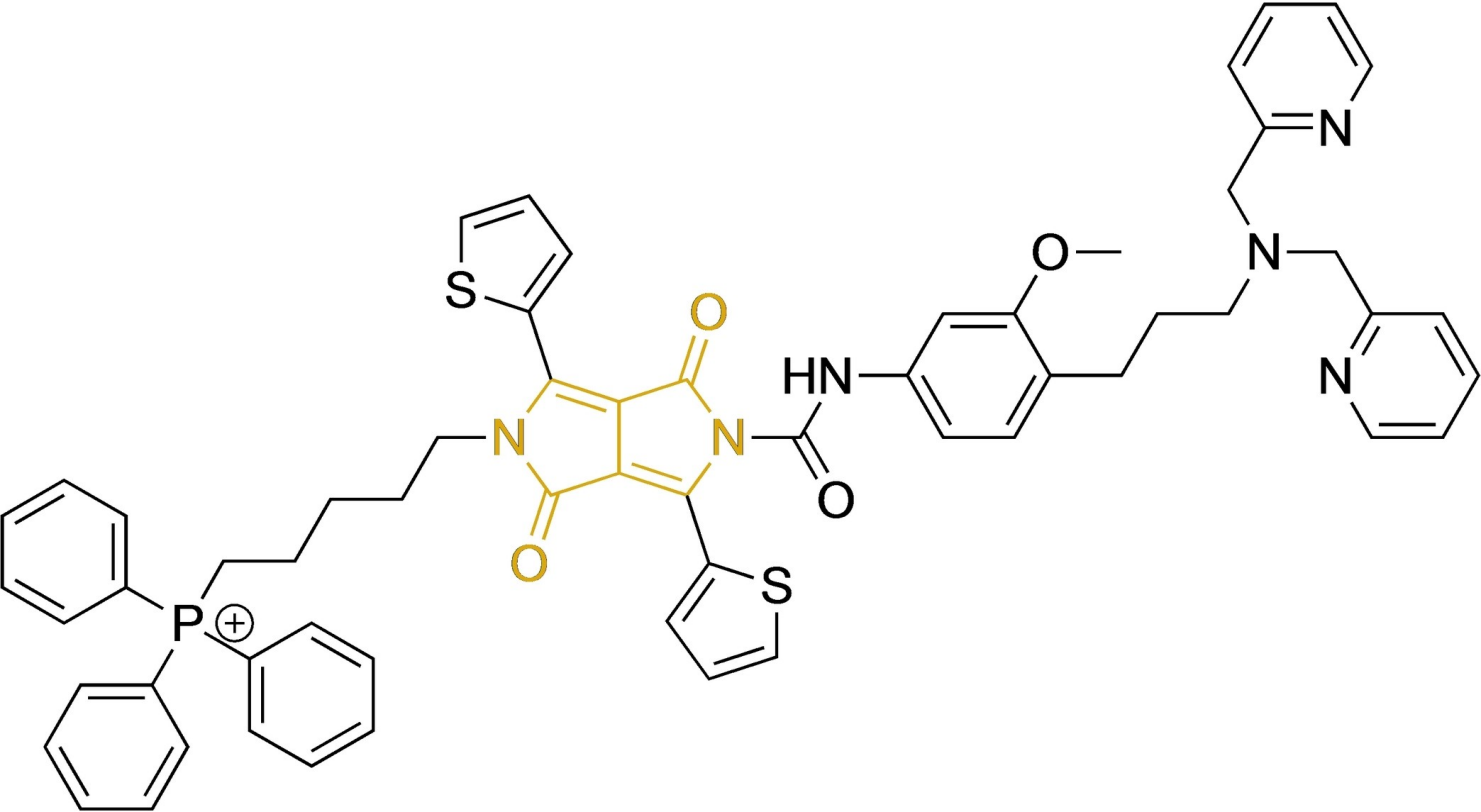	548/565	549	0.02	DMSO
**122**	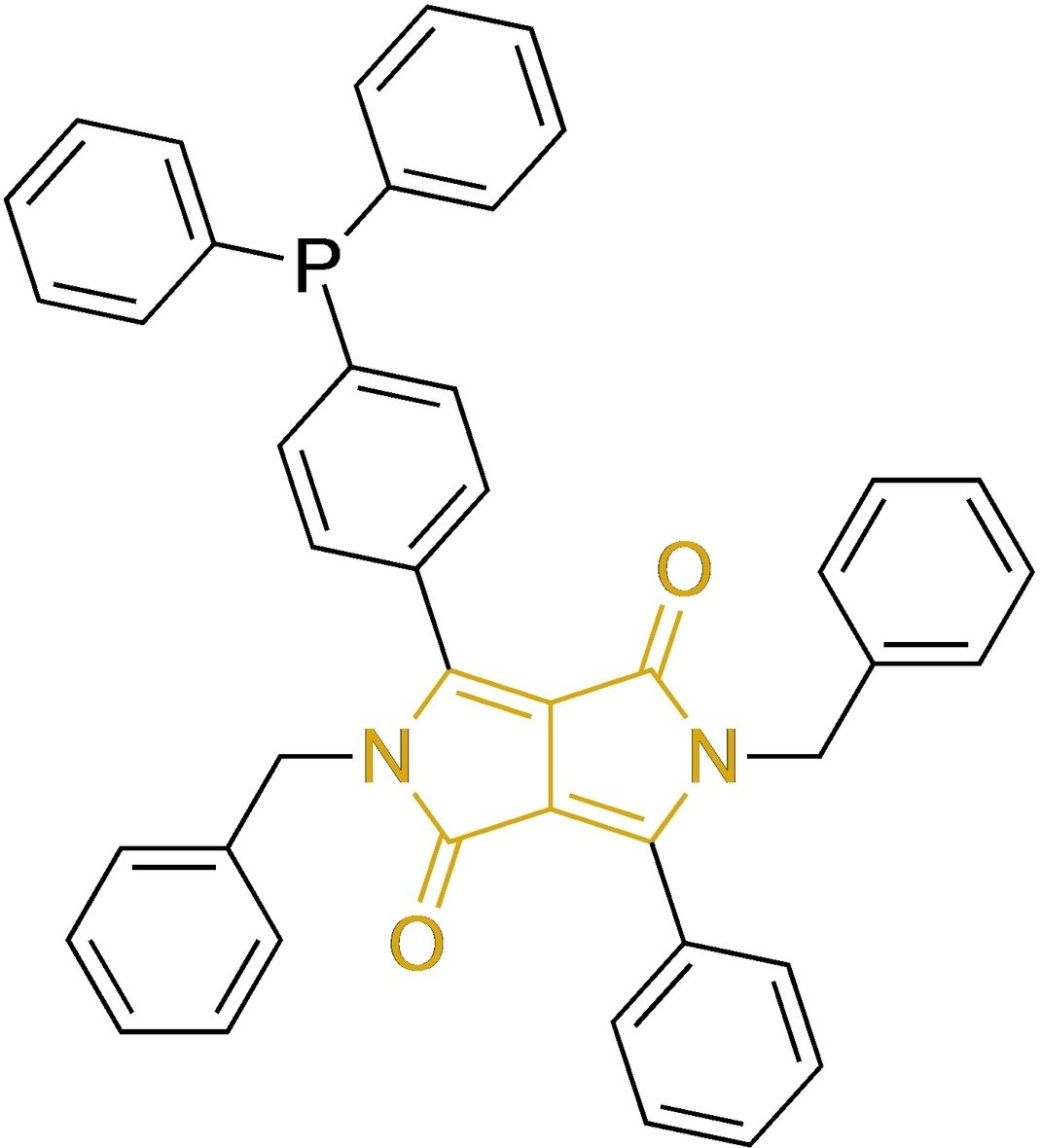	477/549	2749	0.23	DMSO
**123**	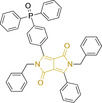	475/550	2870	>0.95	DMSO

It should be noted that similarly to the case for other commonly used scaffolds reviewed herein, DPP chemistries were often conceptualized as to report on the presence of target analytes via so‐called turn‐on effects and thus, their intrinsic quantum efficiency is very low.[^154^, ^155^, ^156^, ^159^] Nonetheless, these can still be very useful in informing future design given that their low φ_f_ can be readily ascribed to efficient intramolecular photoinduced electron transfer processes which are facilitated by atoms bearing lone pair of electrons, such as nitrogen and phosphorous.[^35^, ^154^] As such, said processes can be disrupted by engaging these electrons in covalent bonds (see below). In relation to mitochondrial targeting, reported DPP‐based materials exploit the utilization of commonly employed delocalized lipophilic cations such as triphenylphosphonium and pyridinium.[^154^, ^155^, ^156^, ^159^] Interestingly, both **117** and **118**, encompassing pyridinium units were designed to achieve mitochondrial accumulation with identical asymmetric core rings and N‐substitution.[^154^, ^159^] Despite their different terminal substitutions on the pyridinium nitrogen atom, both were reported to exhibit almost identical photophysical properties (λ_abs/em_^max^=520/652 and 520/651 nm for **117** and **118** in ethanol/PBS, respectively). This reinforces the previous observation regarding the effect that peripheral substitutions on the core rings have on these properties.

**Figure 4:**
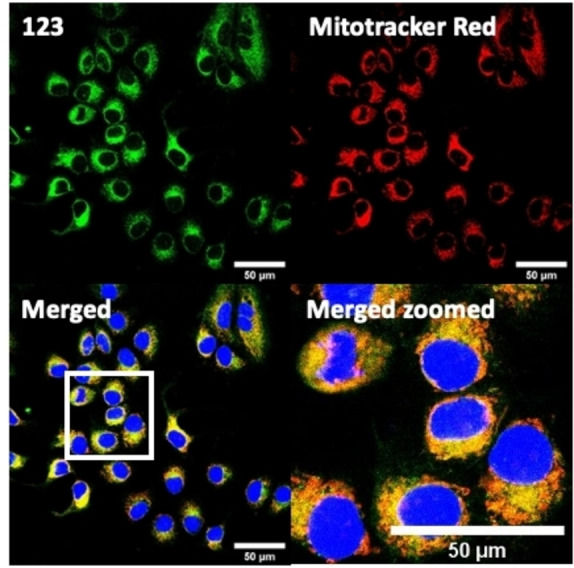
MCF7 intracellular distribution of **123** (250 nM, 0.163 μg mL^−1^) and Mitotracker Red (250 nM, 0.133 μg mL^−1^). Scale bar represents 50 μm. Adapted with permission.^[35]^ Copyright 2022, Wiley‐VCH.

There have been attempts to try and access this target subcellular organelle with neutral DPPs.[^154^, ^157^] However, it should be noted that in some cases these bear very basic nitrogen atoms which are likely protonated in cellular media. Firstly, probe **119**, bearing pyridine core rings and intramolecularly quenched fluorescence due to morpholine moieties (φ_f_=<0.01 in DCM) was reported to exhibit mitochondrial accumulation.^[157]^ However, the results were inconclusive and the observed behavior was attributed to an even distribution of **119** in the cell and exhibited emission occurring in organelles characterized by slightly basic environment rather than actual preferential mitochondrial accumulation. Shortly after, **120** was synthetized to attempt the challenge of mitochondrial accumulation employing neutral probes.^[154]^ This neutral asymmetric probe bearing thiophene core rings was observed not to exhibit cellular uptake. This was successfully reversed by utilizing a triphenylphosphonium targeting unit in the N‐substituted analogue (**121**). More recently, it was demonstrated that selective mitochondrial targeting can be afforded in the absence of delocalized lipophilic cations (Figure 4).^[35]^ Whilst a neutral triphenylphosphine‐bearing DPP (**122**) was characterized by negligible cellular uptake, its oxidized counterpart (**123**) was observed to selectively accumulate within the target organelle at the nanomolar level, with a performance comparable to commercially available mitochondrial staining agents. In addition, the intramolecularly quenched fluorescence in **122** was reversed in **123** by engaging the phosphine lone pair of electrons upon chemical oxidation, affording a remarkably high quantum efficiency (φ_f_=0.23/0.08 and >0.95/0.56 for **122** and **123** in DMSO/H2O (0.5 % DMSO), respectively).^[35]^ This opened up the possibility of further developing superior materials without the risk of negatively impacting on mitochondrial transmembrane potential which is key for the working function of the organelle and represents a significant breakthrough in the field.

## Pyrenes

The planar and rigid polycyclic aromatic motif denoted by pyrene (Figure 2) has been widely exploited as a result of its electronic and photophysical properties.^[160]^ Nonetheless, this interesting core motif is still believed to remain underexploited in the bioimaging field, which can be largely attributed to the lack of a well‐known and clear synthetic methodology for small molecular architectures bearing this core motif.^[161]^ Along these lines, one could think that the simplest approach would be the direct peripheral functionalization of pyrene itself. However, reported methods to do so are rather limited.^[160]^ Electrophilic aromatic substitutions on positions 1, 3, 6 and 8 are possible as these denote the most electron‐rich locations.^[162]^ Nonetheless, the purification of tetrasubstituted pyrenes is often challenging due to their insolubility. Interestingly, monosubstituted pyrenes have also been synthesized from the parent core motif, where further peripheral functionalization can be access under, for example, Heck coupling conditions of relevant bromides with styrenes.^[163]^ As a result, successful approaches often rely on the utilization of non‐pyrene precursors whereby the conjugated core is only accessed at a late synthetic stage. Such so‐called indirect strategies facilitate the realization of a wider range of substituted pyrenes with desired functionalization, such as i) the tetrahydropyrene method to perform electrophilic aromatic substitutions on positions 2 and 7,[^164^, ^165^] ii) the hexahydropyrene approach towards the synthesis of pyrene architectures with fused rings^[166]^ or iii) the construction of pyrenes from biphenyl systems.^[167]^ The latter denotes a highly promising methodology due to the availability of required starting materials.^[161]^

In most cases, reported pyrene‐based systems utilize pyridinium delocalized lipophilic cations as mitochondrial targeting units. In all cases, successful accumulation was observed which denotes a significant superior performance of pyrene materials when compared to other chemistries, such as cyanines, which require the presence of additional delocalized lipophilic cations (see above). Probes **124** ^[168]^ and **125** ^[169]^ (, Table 5) bear terminal boronate functionalization for sensing application. As such, their arguably low quantum yields (φ_f_=0.07 and 0.27 for **124** and **125** in DMSO and sodium borate buffer, respectively) are intended for this purpose. Removal of the carbonyl group on progression from **124** to **125** results in a significant bathochromic shift (λ_abs/em_^max^=435/555 and 440/600 nm for **124** and **125** in DMSO and sodium borate buffer, respectively) as well as an increase in the quantum efficiency (see above). This is particularly noteworthy given the φ_f_ for **125** was determined in water, where lower efficiencies are expected, associated to accessible radiationless deactivation pathways due to aggregation.

**Table 5:** Chemical structure, absorption and fluorescent emission maxima (λ_abs/em_^max^/nm), Stokes shifts (Δλ/cm^−1^), fluorescence quantum yield (φ_f_) and solvent use for the characterization of reported pyrene‐based architectures.

Molecule	Chemical structure	λ_abs/em_^max^/nm	Δλ/cm^−1^	φ_f_	Solvent
**124**	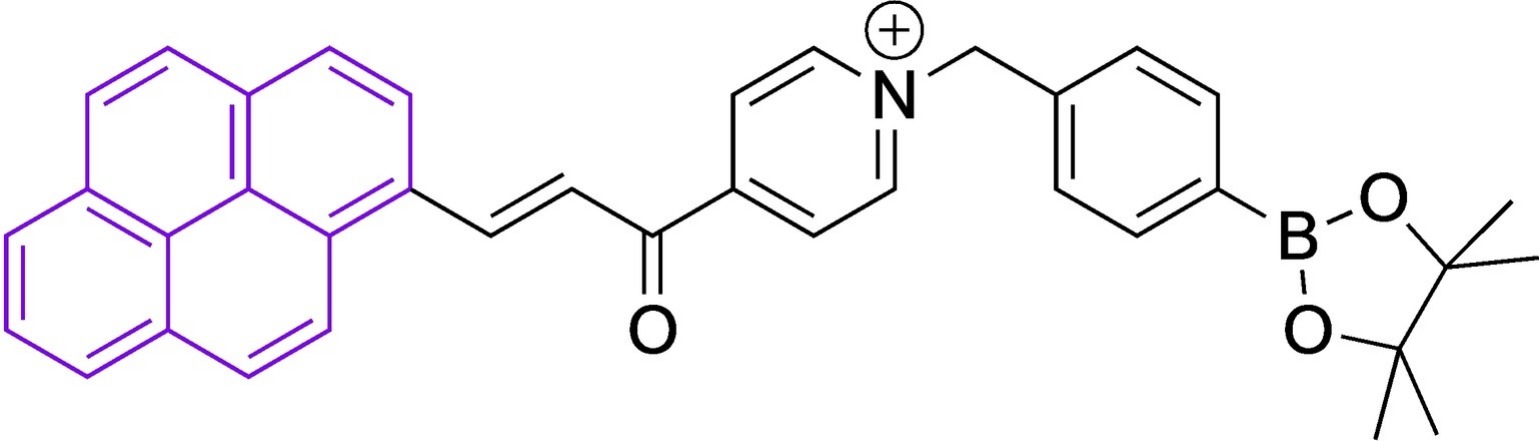	435/555	4970	0.07	DMSO
**125**	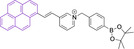	440/600	6060	0.27	Sodium borate buffer (20 % DMSO)
**126**	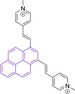	452/624	6098	0.72	DMSO
**127**	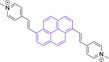	480/619	4678	0.82	DMSO
**128**	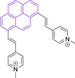	477/616	4730	0.76	DMSO
**129**	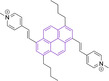	510/650	4223	0.80	DMSO
**130**	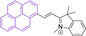	354/620	12119	‐	PBS buffer (40 % DMSO)

The use of pyridinium motifs conjugated to the pyrene core via styryl substitutions has been significantly exploited. As such, systems bearing two styrylpyridinium moieties were synthesized, differentiated by the relative position of these functional groups.^[170]^ It was observed that irrespective of the substitution pattern, 1,3 (**126**), 1,6 (**127**) or 1,8 (**128**) all probes were characterized by large quantum efficiencies (φ_f_>0.72 in DMSO). Progression from **126** to **127** and **128** was observed to result in bathochromic shifts in their absorption spectra. Addition of n‐butyl chains on positions 3 and 8 of **127** in probe **129** was observed to induce a bathochromic shift while maintaining the high quantum yield (λ_abs/em_^max^=480/619 and 510/650 nm for **127** and **129** in DMSO, respectively).^[171]^ Compound **130** was synthesized with indolium and not pyridinium targeting motif, which is conjugated to the core by a styryl system.^[172]^ In agreement with observations reported for cyanine based materials, where this functionality is ubiquitous, the probe did not exhibit selective but only preferential mitochondrial accumulation.

## Xanthenes

This class of materials encompasses widely known and exploited organic scaffolds such as rhodamines, fluoresceins and eosins.[^54^, ^173^, ^174^] The core structure (Figure 2) is inherently photostable and characterized by attractive photophysical properties for bioimaging such as high brightness resulting from both large quantum yields as well as large absorption factors.[^173^, ^174^] However, peripheral substitutions have the ability to critically modify such properties, with significant photodegradation following continuous irradiation being reported. Along those lines, replacing the carbonyl oxygen for nitrogen on position 6 of the core motif on progression from fluorescein to rhodamine, also carries an associated diminishment in the photostability of the material. Thus, rhodamines are often considered to be more suitable for continuous exposure to excitation sources and confocal imaging work than fluoresceins.^[175]^ Structural modifications are also associated to the decrease in quantum yields observed in substituted xanthenes through efficient photoinduced electron transfer processes from electron rich substituents on position 9 onto the core motif.^[173]^ However, the coplanarity requirement between donor and acceptor for this radiationless transition to be efficient allows for judicious design approaches, such as (i) the use of ortho substitutions to induce out‐of‐plane re‐arrangements and/or (ii) the use of electron deficient groups on that position to lower the electron donating ability.

Xanthene based systems can be synthetically accessed via modifications of the original protocols dating back to the 1880s, such as the ZnCl_2_ or TsOH catalyzed condensation of phthalic or propionic acid with 3‐aminophenol.[^176^, ^177^, ^178^] However, despite this being ubiquitous in the literature, it is known for having significant drawbacks such as harsh reaction conditions, low yields and importantly the difficulty in separating isomers. In response to this, alternative strategies have been proposed. For instance, rhodamine‐based architectures can be made from appropriate fluorescein triflates under Buchwald–Hartwig cross‐coupling conditions.^[173]^ Problems associated to condensation‐based synthesis can also be bypassed by addition of organometallic species to fully N‐alkylated diaminoxanthone. It is of note that xanthenes can be easily accessed under Grignard conditions and subsequent dehydration from functionalized xanthones which are commercially available.[^174^, ^179^]

Preferential mitochondrial targeting in xanthenes has been reported for systems whereby the core oxygen atom is protonated. In regard, **131** (Table 6) was designed bearing diethylamino substitution on position 6, which resembles a widely used design in coumarin‐based systems (see above).^[180]^ Its very low (φ_f_<0.01) quantum efficiency results from the intrinsic fluorescence being intramolecularly quenched by the azo moiety on position 3 for sensing purposes. In fact, replacement of the azo substitution for another diethylamino in **132** increased the quantum yield to 0.39 in PBS.^[181]^ Despite the charged core oxygen atom in this molecule, mitochondrial accumulation was also ensured via pyridinium cation substitution on the amine on position 9. These peripheral motifs resulted in a significant red shift when compared to **131** (λ_abs/em_^max^=365/500 and 590/605 nm for **131** and **132** in PBS buffer, respectively) and small Stokes shift (Δλ=420.23 cm^−1^). Two pyridinium targeting moieties were utilized in **133**. These were linked to the core on positions 3 and 6 via sulfoxide groups. Interestingly, despite the targeting units, the probe exhibited some accumulation within the lysosomes as well as mitochondria. This probe, as many other reported to date exploit rhodol‐based spirocyclic or spirolactam substitution on position 9 of the core. These, which exist as an equilibrium between the closed and open form exhibit critically different photophysical and biological properties. In short, the open zwitterionic form is characterized by poor cellular uptake and good quantum efficiency, whilst the opposite is observed for the closed form.[^173^, ^174^, ^178^] Similar to **133**, **134**–**137** also employed the rhodol‐based spirocyclic moiety on position 9 of the core. **134** was designed with a triphenylphosphonium targeting motif and boronate on position 6 for sensing purposes.^[183]^ Probe **135** and **136** also bear the same terminal mitochondrial targeting substitution, however their photophysical properties were not fully characterized.[^184^, ^185^] It is noteworthy that the neutral counterpart of **136** was observed not to exhibit mitochondrial accumulation. Another neutral rhodol‐based spirocyclic system (**137**) was evaluated, bearing nitroxide groups on the amines on positions 3 and 6 of the core.^[186]^ Mitochondrial accumulation as well as a significant quantum yield increase (φ_f_=0.83 in PBS:DMSO (5 %)) was observed upon photoactivation and cleavage of these nitroxide groups. Target accumulation was also attempted with a neutral probe, **138**.^[187]^ This, which bears diethylamino and spirolactam substitutions on positions 3/6 and 9, respectively was observed to also exhibit some accumulation in the lysosomes. Its structural analogue (**139**), also neutral and with comparable absorption and fluorescence emission maxima, was characterized by a large quantum efficiency (φ_f_=0.51 in ethanol:water, 1 : 9) and to selectively accumulate in the mitochondria. However, both the φ_f_ and accumulation can be associated to the protonation of the molecule at the working pH.^[188]^ Along these lines, pH sensitivity is highly characteristic of xanthene‐based dyes.[^173^, ^174^] Probe **140**, a fluorescein based system with a terminal nitroxide substitution was observed to exhibit modest cellular uptake.^[175]^ Its pyrrolidinium‐charged rhodamine‐based analogues, **141–142** were characterized by both improved targeted accumulation and photostability as a result of the different substitution.^[175]^ Despite the poor photophysical characterization, the choice of channels indicates a hypsochromic shift on going from **141** to **142**, which can be attributed to the selection of ethyl instead of methyl substitutions, respectively. Interestingly, probe **142** was observed to exhibit highly desired two‐photon absorption at 820 nm. Another rhodamine‐based xanthene bearing pyrrolidinium targeting moiety on position 6 was reported. This probe (**143**) and its morpholinium counterpart (**144**) were observed to accumulate well within the mitochondria and to exhibit spectral maxima in line with other derivatives (λ_abs/em_^max^=563/580 and 553/578 nm for **143** and **144** in phosphate buffer, respectively).^[189]^ Their low quantum yields can be attributed to non‐protonated phenylic carboxylic groups.[^173^, ^174^]

**Table 6:** Chemical structure, absorption and fluorescent emission maxima (λ_abs/em_^max^/nm), Stokes shifts (Δλ/cm^−1^), fluorescence quantum yield (φ_f_) and solvent use for the characterization of reported pyrene‐based architectures.

Molecule	Chemical structure	λ_abs/em_^max^/nm	Δλ/cm^−1^	φ_f_	Solvent
**131**	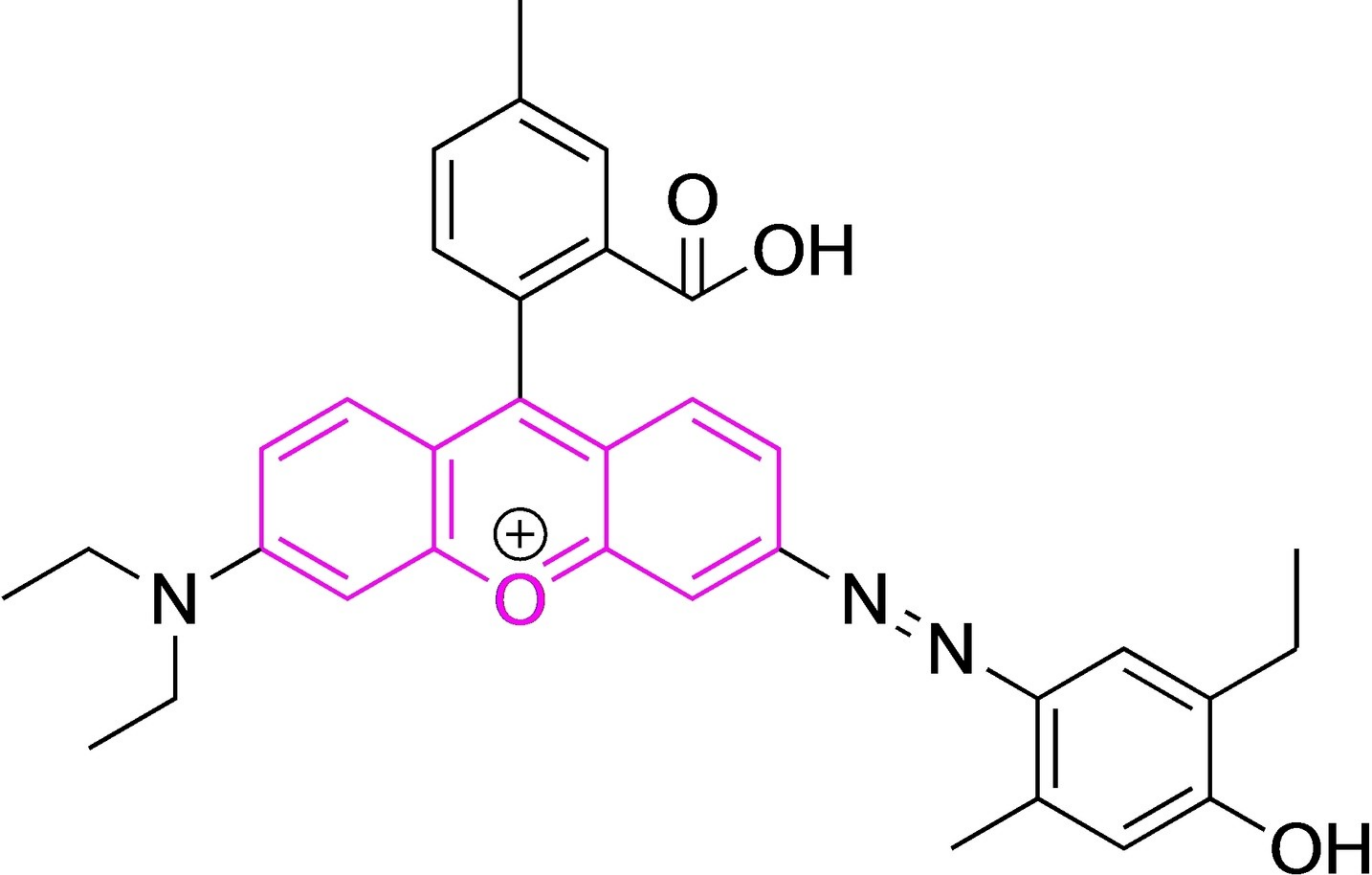	365/500	7397	<0.01	PBS
**132**	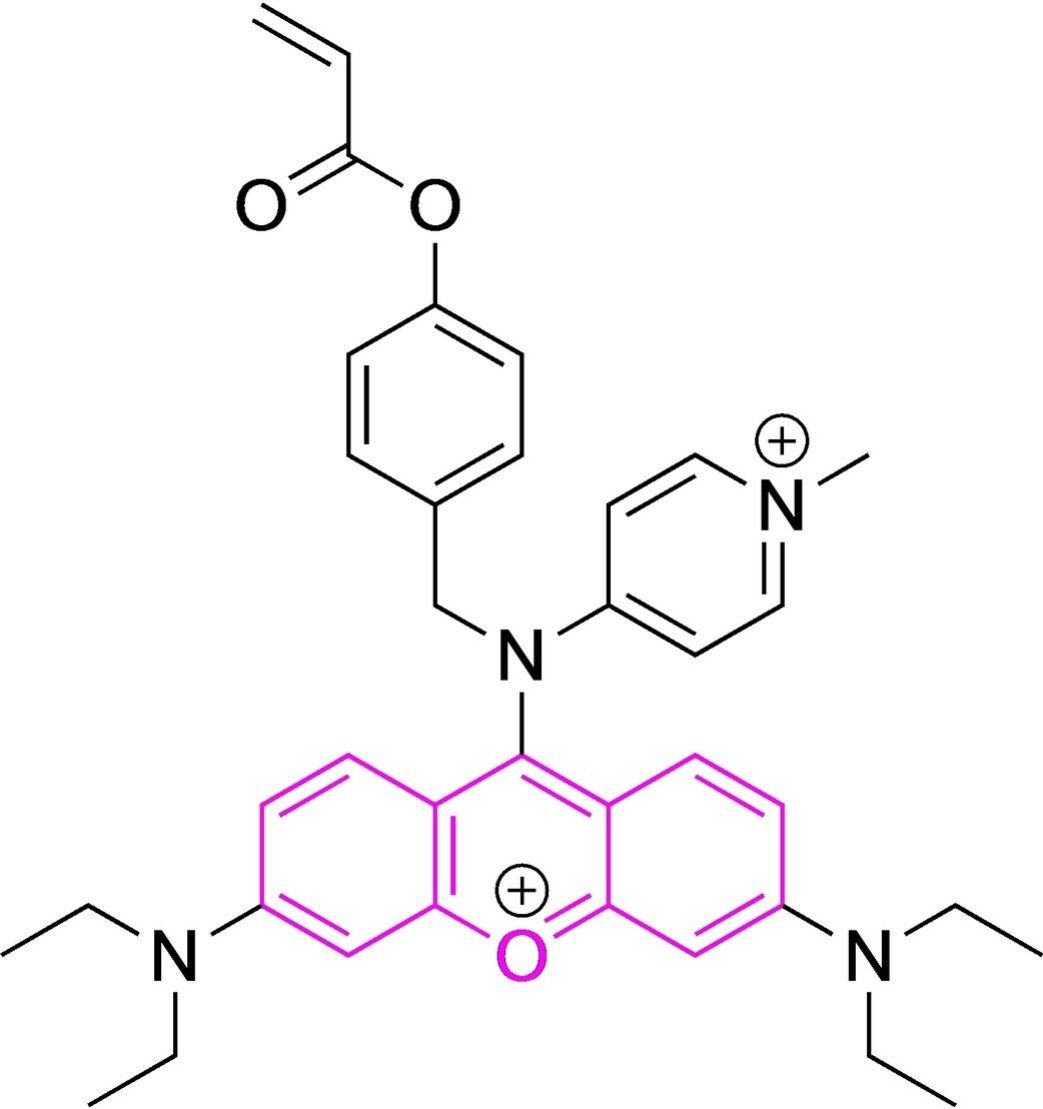	590/605	420	0.39	PBS
**133**	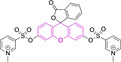	494/514	787	–	PBS
**134**	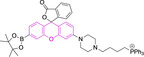	510/540	1089	0.02	HEPES buffer
**135**	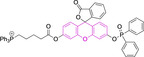	291/515	14946	0.06	PBS
**136^[a]^**	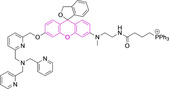	–/550	–	–	HEPES buffer
**137**	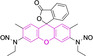	520/550	1048	0.83	PBS:DMSO (5 %)
**138**	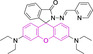	530/578	1566	–	PBS
**139**	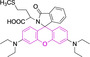	563/585	667	0.51	Ethanol/water (1 : 9)
**140**	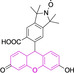	–	–	–	–
**141**	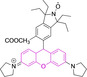	–	–	–	–
**142**	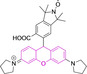	–	–	–	–
**143**	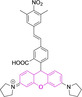	563/580	520	0.05	PBS (1 % DMSO)
**144**	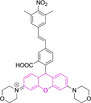	553/578	782	0.03	PBS (1 % DMSO)
**145**	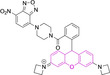	565/585	605	0.17	PBS
**146**	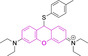	590/620	820	–	PBS
**147**	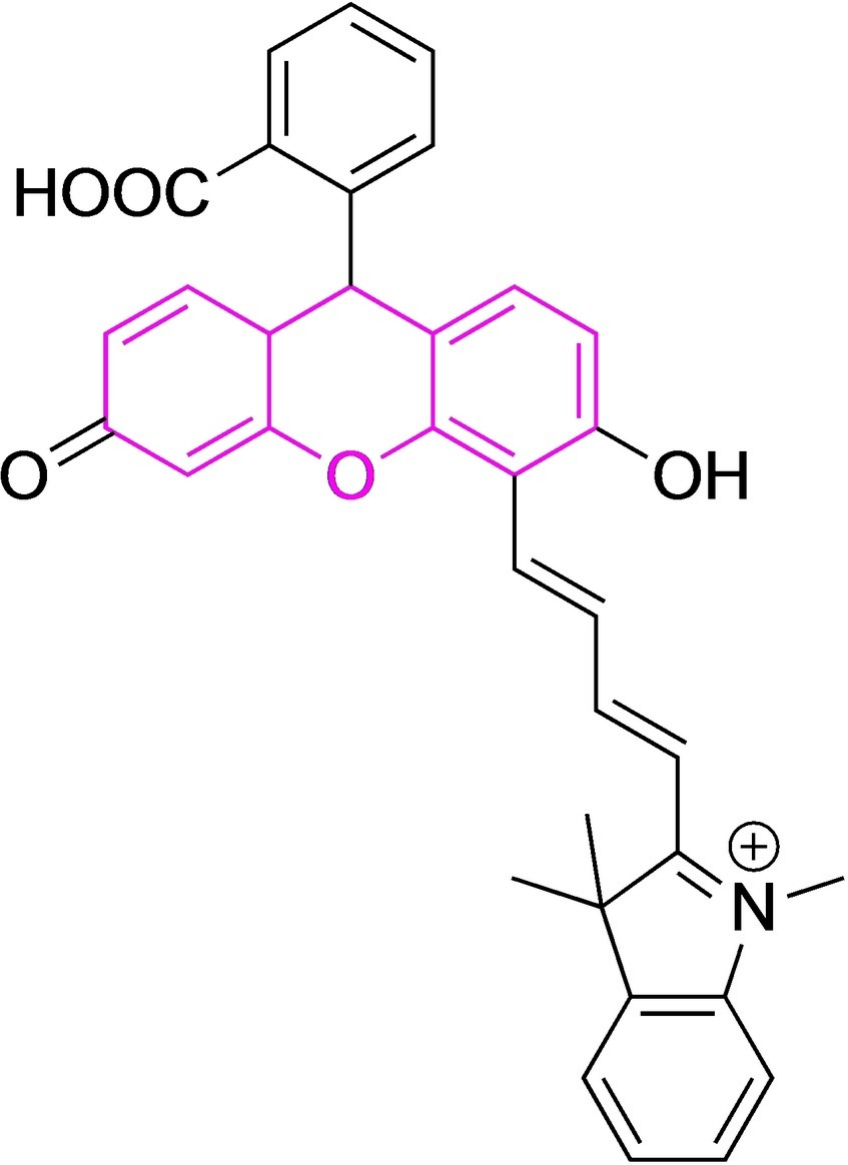	475/566	3384	0.05	DMSO

^[a]^ Absorption and fluorescence emission maxima approximated from spectra.

Substitutions on the phenylic acid group in **145** to yield ketones were observed to alleviate the quantum efficiency (φ_f_=0.05 and 0.17 for **143** and **145** in phosphate buffer, respectively) whilst maintaining a similar core structure with azetidinium instead of pyrrolidinium moieties.^[190]^ A relatively simpler structure (**146**) bearing diethylammonium and thiophenyl moieties on position 3 and 9 was reported to be bathochromically shifted when compared to previous rhodamines (e. g. λ_abs/em_^max^=553/578 and 590/620 nm for **144** and **146** in phosphate buffer, respectively).^[191]^ Lastly, **147** exploits a different design in a fluorescein‐based xanthene.^[192]^ Mitochondrial accumulation is facilitated through indazolium substitution on previously unexploited position 4, which is conjugated to the core via styryl motif. The large Stokes shift (Δλ=3384.79 cm^−1^) can be associated to the flexibility of the latter and the greater number of conformers accessed.

## Summary and Outlook

The realization of novel organic conjugated fluorophores with specific subcellular accumulation has attracted an increasingly large body of interest in recent years, with more than 70 % of all architectures being reported in the last five years. In doing so, a number of chemistries have been exploited, namely BODIPYs, coumarins, cyanines, diketopyrrolopyrroles, pyrenes and xanthenes. In some cases, such as cyanine‐ and, to a lesser extent xanthene‐based platforms, mitochondrial accumulation can be facilitated without a requirement for peripheral substitutions bearing delocalized lipophilic cations. In turn, charged motifs within their core structures can function as mitochondrial targeting units. Interestingly, whilst selective accumulation can be induced in BODIPYs and diketopyrrolopyrroles by incorporating a single cationic moiety, the observed scenario in cyanines is somehow different. This class of materials, as well as others, such as pyrenes were observed to only achieve preferential and not selective accumulation bearing indolium and pyridinium targeting motifs. Interestingly, the use of neutral structures whilst maintaining selective subcellular organelle targeting has been demonstrated for BODIPYs, coumarins as well as diketopyrrolopyrroles. This denotes a critical element in the design of novel platforms in light of the associated detrimental effects in mitochondrial working function when charged probes are employed. Thus, recent efforts indicate that judicious choice of uncharged peripheral alternations can determine cellular uptake and accumulation and not only the photophysical properties. In most cases, molecules conforming to the reviewed core motifs are characterized by good thermal and photostability. However, design strategies must be carefully considered in the case of cyanine‐ and xanthene‐based architectures. Whilst the extension of the polymethine chain in the former is widely exploited towards achieving bathochromic spectral shifts, this approach also carries an associated detrimental effect on the stability of the probes. Heptamethine chains represent a compromise and are ubiquitous as a result. Continuous irradiation can also lead to a diminishment in the stability of xanthenes when the carbonyl oxygen in position 6 of the core is substituted for nitrogen on progression from fluorescein to rhodamine. Thus, rhodamines are often considered to be more suitable for confocal work and continuous exposure to excitation sources than their fluorescein analogues. Coumarin and pyrene scaffolds are intrinsically blue shifted when compared to other core motifs investigated herein. In light of this, performing peripheral modifications is essential to facilitate their use with longer excitation wavelength irradiation sources. In addition, these structural changes in the case of coumarins carry the added benefit of increasing their Stokes shifts. A drawback associated to the utilization of BODIPYs and cyanines is their intrinsically narrow Stokes shifts which warrants the introduction of flexible peripheral substituents to induce greater structural rearrangements upon photoexcitation. Narrow Stokes shifts often associated to diketopyrrolopyrrole architectures are nonetheless a consequence of the utilization of thiophene and furan core rings and are not observed in their phenyl substituted counterparts which represent superior alternatives for bioapplications. Despite the suitability of probes often being evaluated solely on their fluorescence quantum yields, their brightness should be considered instead. Along these lines, it is important to note that whilst both cyanine‐ and coumarin‐based systems are in most cases characterized by arguably low quantum yields, the brightness in the case of the former is high due to large absorption factors. Fluorescence emission efficiencies in BODIPY and xanthene systems can be detrimentally impacted by substitutions on positions 8 and 9, respectively which foster access to radiationless deactivation mechanisms. These can be judiciously prevented by design strategies whereby out‐of‐plane re‐arrangements are facilitated by ortho substitutions that prevent coplanarity requirements for such radiationless decay pathways. Despite significant efforts, we consider that there is an unmet need of studies whereby structural analogues characterized by small systematic substitutions are evaluated. This is believed to be particularly warranted in relation to the biological evaluation of reported architectures, given that in most cases complete characterization and assessment of subcellular accumulation following cell uptake is solely limited to those molecules that anticipate appropriate performance on preliminary evaluations. These approaches limit critical information that can guide future design strategies, irrespective of the chemistries used. Similarly, we consider that thorough photophysical characterization is essential in understanding end performance of synthesized materials. Such considerations would aid in paving the way for the development of the next generation of small organic fluorophores for mitochondrial bioimaging, which could include utilization of currently underexploited chemistries such as diketopyrrolopyrroles and pyrenes in addition to novel core motifs. As a result, we anticipate this work to guide the development of the next generation of mitochondrial small molecule fluorescent platforms by enhancing multidisciplinary awareness on existing efforts and fostering collaborative work among chemists and cell biologists.

## Conflict of interest

The authors declare no conflict of interest.

1

## Biographical Information

*Hannah Crawford graduated with a degree in Chemistry from the University of Kent before continuing her studies with an MSc by Research at the same Institution, under the supervision of Dr Christopher Serpell. In 2021 she moved to the University of Hertfordshire to undertake a PhD on the development of small molecule fluorophores for mitochondrial bioimaging*.

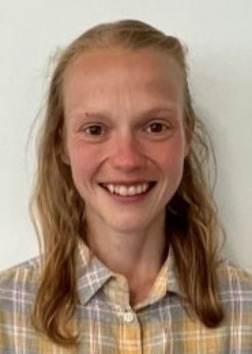

## Biographical Information

*Maria Dimitriadi is a Reader in Molecular Genetics at the University of Hertfordshire. She uses the nematode Caenorhabditis elegans to understand the molecular and cellular mechanisms that underlie neurodegenerative disorders with a particular emphasis on spinal muscular atrophy, a devastating childhood motor neuron disease*.

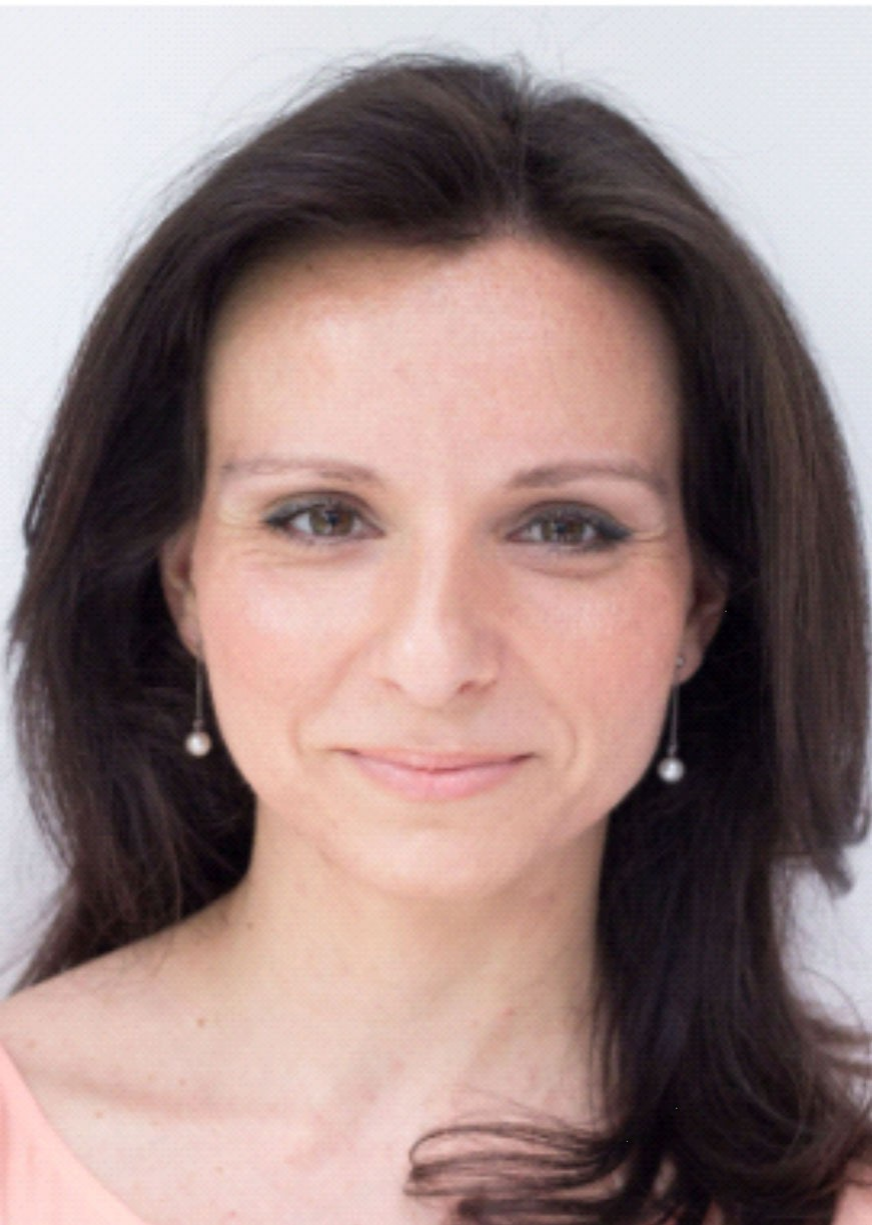

## Biographical Information

*Jatinder Bassin is a Principal Lecturer in Pharmaceutical Chemistry at the University of Hertfordshire. His research interests are in the synthesis of novel heterocyclic compounds as well as molecules against a number of biological targets, including antioxidants and antibacterial agents. Other areas under investigation are the isolation, structure elucidation and synthesis of natural products of pharmacological interest*.

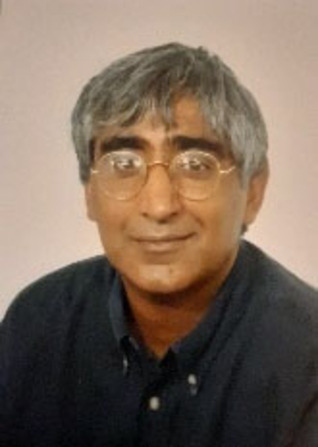

## Biographical Information

*Michael Cook is a Reader in Thermoresponsive Polymers at the University of Hertfordshire. His Research interest is in the synthesis of temperature‐responsive polymers and probing links between nanoscale and bulk phenomena*.

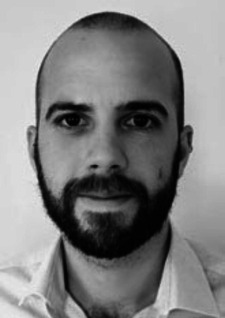

## Biographical Information

*Thais Fedatto Abelha is a Lecturer in Therapeutic Chemistry at the Universitat de Barcelona. Her research interest is in the development of light‐activated materials with applications in diagnosis and treatment*.

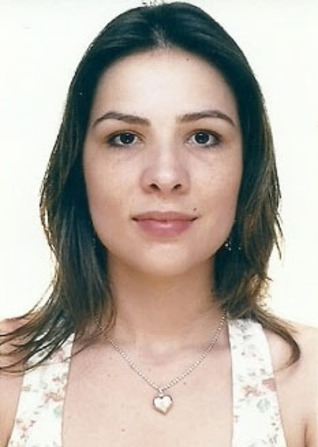

## Biographical Information

*Jesus Calvo‐Castro is a Senior Lecturer in Pharmaceutical Chemistry at the University of Hertfordshire. His research interests and expertise are on the development of novel organic‐based materials that can be exploited in optoelectronic applications, ranging from charge transfer mediators to detection of small molecule analytes as well as preferential bioimaging of subcellular organelles*.

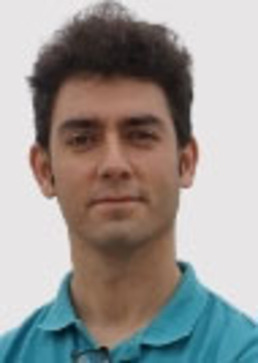

## Data Availability

The data that support the findings of this study are available from the corresponding author upon reasonable request.
